# A broadly neutralizing antibody against the SARS-CoV-2 Omicron sub-variants BA.1, BA.2, BA.2.12.1, BA.4, and BA.5

**DOI:** 10.1038/s41392-024-02114-6

**Published:** 2025-01-13

**Authors:** Zhe Chen, Leilei Feng, Lei Wang, Li Zhang, Binyang Zheng, Hua Fu, Fengdi Li, Ligai Liu, Qi Lv, Ran Deng, YanLi Xu, Yongfeng Hu, Jianhua Zheng, Chuan Qin, Linlin Bao, Xiangxi Wang, Qi Jin

**Affiliations:** 1 NHC Key Laboratory of Systems Biology of Pathogens, State Key Laboratory of Respiratory Health and Multimorbidity, National Institute of Pathogen Biology, and Center for Tuberculosis Research, Chinese Academy of Medical Sciences & Peking Union Medical College, Beijing, 100730 China; 2 CAS Key Laboratory of Infection and Immunity, National Laboratory of Macromolecules, Institute of Biophysics, Chinese Academy of Sciences, Beijing, 100101 China; 3 University of Chinese Academy of Sciences, Beijing, 100049 China; 4 Department of Vaccine Clinical Evaluation, Jiangsu Provincial Center for Disease Prevention and Control, 172 Jiangsu Road, Gulou Qu, Nanjing, 210009 China; 5 Institute of Laboratory Animal Sciences, Chinese Academy of Medical Sciences (CAMS) and Comparative Medicine Center, Peking Union Medical Collage (PUMC), Key Laboratory of Human Disease Comparative Medicine, Ministry of Health, Beijing, China; 6 Beijing Ditan Hospital, Capital Medical University, Beijing, 100015 PR China; 7 Chronic Disease Management Center, Beijing Ditan Hospital, Capital Medical University, Beijing, 100015 PR China

**Keywords:** Infectious diseases, Infectious diseases

## Abstract

The global spread of Severe Acute Respiratory Syndrome Coronavirus 2. (SARS-CoV-2) and its variant strains, including Alpha, Beta, Gamma, Delta, and now Omicron, pose a significant challenge. With the constant evolution of the virus, Omicron and its subtypes BA.1, BA.2, BA.3, BA.4, and BA.5 have developed the capacity to evade neutralization induced by previous vaccination or infection. This evasion highlights the urgency in discovering new monoclonal antibodies (mAbs) with neutralizing activity, especially broadly neutralizing antibodies (bnAbs), to combat the virus.In the current study, we introduced a fully human neutralizing mAb, CR9, that targets Omicron variants. We demonstrated the mAb’s effectiveness in inhibiting Omicron replication both in vitro and in vivo. Structural analysis using cryo-electron microscopy (cryo-EM) revealed that CR9 binds to an epitope formed by RBD residues, providing a molecular understanding of its neutralization mechanism. Given its potency and specificity, CR9 holds promise as a potential adjunct therapy for treating Omicron infections. Our findings highlight the importance of continuous mAb discovery and characterization in addressing the evolving threat of COVID-19.

## Introduction

The world has been ravaged by the Severe Acute Respiratory Syndrome Coronavirus 2 (SARS-CoV-2), the cause of the COVID-19 pandemic declared by the World Health Organization. As a positive-strand ribonucleic acid (RNA) virus, SARS-CoV-2 inherently possesses the capacity to mutate and antigenically vary.^1^ Variants of Concern (VoC), such as Alpha, Beta, Gamma, Delta, and Omicron, have emerged intermittently. Omicron, first identified in Botswana in November 2021, quickly escalated to become the predominant strain.^2^ The unprecedented speed of Omicron’s and its sub-variants BA.1, BA.2, BA.3, BA.4, and BA.5’s evolution allows them to evade the neutralizing antibodies triggered by prior vaccination or infection, posing a significant challenge.^3^

Certain mutations can confer fitness advantages by enhancing heritability or bypassing humoral immune responses triggered by vaccination or natural infection.^4^ The Omicron sub-variants BA.1, BA.2, and BA.5 of the novel coronavirus exhibit remarkable neutralization and evasion capabilities, thereby posing significant challenges to the effectiveness of humoral immunity.^5,6^ Recent studies suggest that these sub-variants may evade a broader range of antibodies in the bloodstream induced by vaccination or prior infection, potentially enhancing their resistance to antiviral and monoclonal antibody (mAb) drug therapies.^7^

Amid the COVID-19 pandemic, intensive research has been conducted on the creation of monoclonal antibodies (mAbs) for treating and preventing SARS-CoV-2 infections. The receptor binding domain (RBD) of the spike protein, which interacts with the host’s angiotensin-converting enzyme 2 (ACE2) receptor, has emerged as the primary focus for powerful neutralizing antibodies.^8^ A promising treatment approach involves targeting highly conserved epitopes with minimal mutation potential. Such epitopes, whose mutation incurs high fitness costs for pathogens, could potentially eliminate any selection advantage, offering a promising avenue for effective therapeutic development.^9^

The ongoing immune evasion strategies employed by SARS-CoV-2 mutations underscore the critical need for continuous research on novel sets of monoclonal antibodies (mAbs) with neutralizing activity, particularly broadly neutralizing antibodies (bnAbs). In our current study, we characterized a fully human neutralizing mAb called CR9 against the Omicron variants. This mAb was identified through screening a Fab antibody-phage library derived from a recovered donor who had survived a SARS-CoV-2 infection. We characterized the neutralization and binding properties of CR9 in vitro, employing structural studies to pinpoint its epitopes on the virion. Furthermore, we conducted in vivo studies to investigate its efficacy in preventing and treating SARS-CoV-2 infection in mice. Our results demonstrated that CR9 mAb completely inhibited Omicron replication in vitro. Cryo-electron microscopy (cryo-EM) revealed that the epitope recognized by CR9 is composed of residues from the virus’s spike protein RBD in complex with the Fab fragment. In mouse models, CR9 exhibited effective protection against Omicron challenges, both prophylactically and therapeutically, significantly reducing virus titers. Therefore, CR9 holds promise as a potential adjunctive therapy for the treatment of Omicron infections.

## Results

### Characterization of the neutralizing mAb CR9 against Omicron

**Fig. 1:**
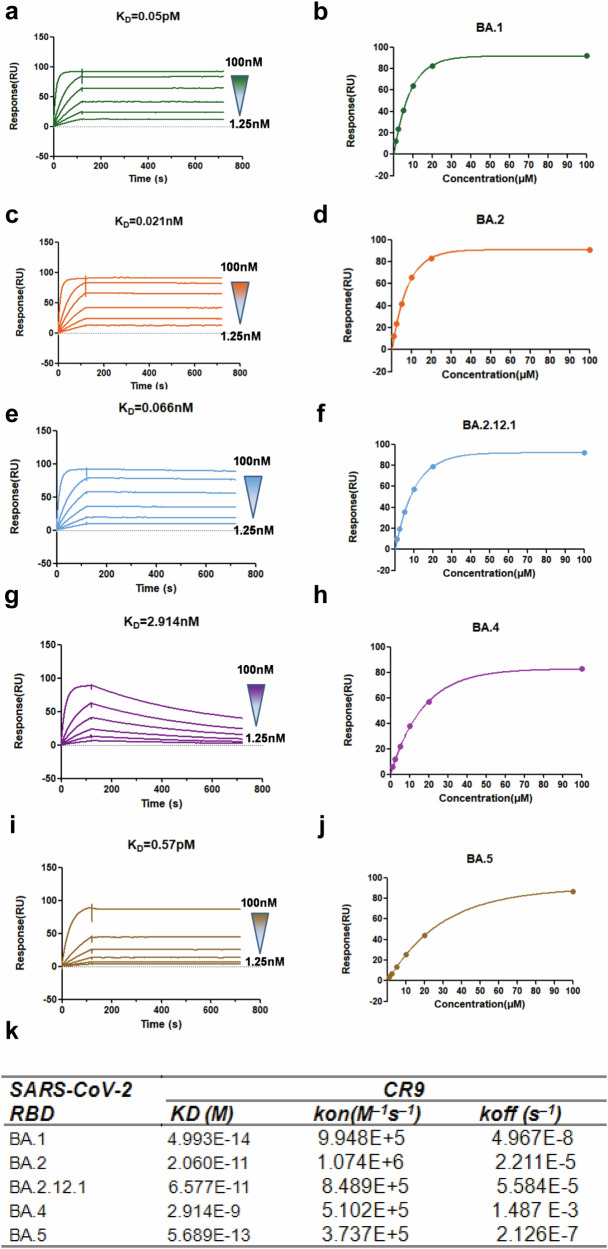
CR9 binding to SARS-CoV-2 RBD measured using SPR. Profiles are shown for BA.1 (**a**), BA.2 (**c**), BA.2.12.1 (**e**), BA.4 (**g**), and BA.5 (**i**). CR9 binding to SARS-CoV-2 RBD measured using KD curve are shown for BA.1 (**b**), BA.2 (**d**), BA.2.12.1 (**f**), BA.4 (**h**), and BA.5 (**j**). **k** Summary of SPR kinetic and affinity measurements. The equilibrium dissociation constant (KD), association constant (kon), and dissociation constant (koff) are presented

**Fig. 2:**
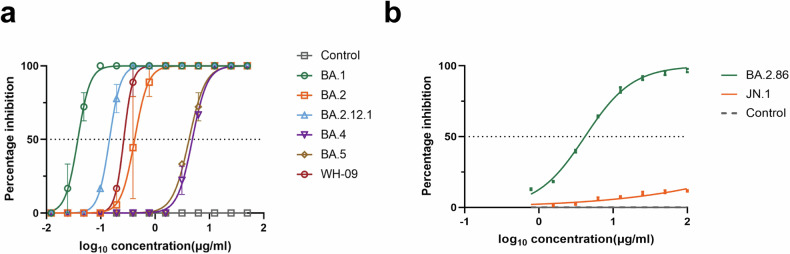
Neutralizing activity of CR9 against SARS-CoV-2. **a** Neutralizing abilities of antibody CR9 against the five Omicron sub-variants BA.1, BA.2, BA.2.12.1, BA.4, BA.5 and wild type(WH-09) in vitro. Irrelevant human immunoglobulin G (IgG) was used as a control. **b** Neutralizing abilities of antibody CR9 against the two pseudoviruses Omicron sub-variants BA.2.86 and JN.1. in vitro. Pseudoviruses HIV was used as a control

A Fab phage display library comprising 1 × 10^8^ independent clones was established. After three rounds of panning, a Fab antibody showing robust reactivity with antigen CR9 was isolated and subsequently transformed into a full-length human IgG1, encompassing light chain *VK3* and heavy chain *VH3*. Affinity measurements revealed dissociation constant values of the CR9 antibody against various Omicron sub-variants: BA.1 (4.993E-14), BA.2 (2.060E-11), BA.2.12.1 (6.577E-11), BA.4 (2.914E-9), and BA.5 (5.689E-13) (Fig. 1a–j). We further evaluated the neutralizing abilities of CR9 against five Omicron sub-variants and the wild-type (WH-09) in vitro. The resulting IC_50_ values were as follows: BA.1 (0.038 µg/ml), BA.2 (0.419 µg/ml), BA.2.12.1 (0.144 µg/ml), BA.4 (4.847 µg/ml), BA.5 (4.214 µg/ml), and WH-09 (0.264 µg/ml) (Fig. 2a). To gain further insights into the neutralization activity of CR9 against recent variants, we generated pseudoviruses for BA.2.86 and JN.1. Notably, CR9 exhibited neutralizing activity against BA.2.86 with an IC_50_ of 4.167 µg/ml but failed to neutralize JN.1(Fig. 2b).

### Prophylactic and therapeutic effects of CR9 in mice

**Fig. 3:**
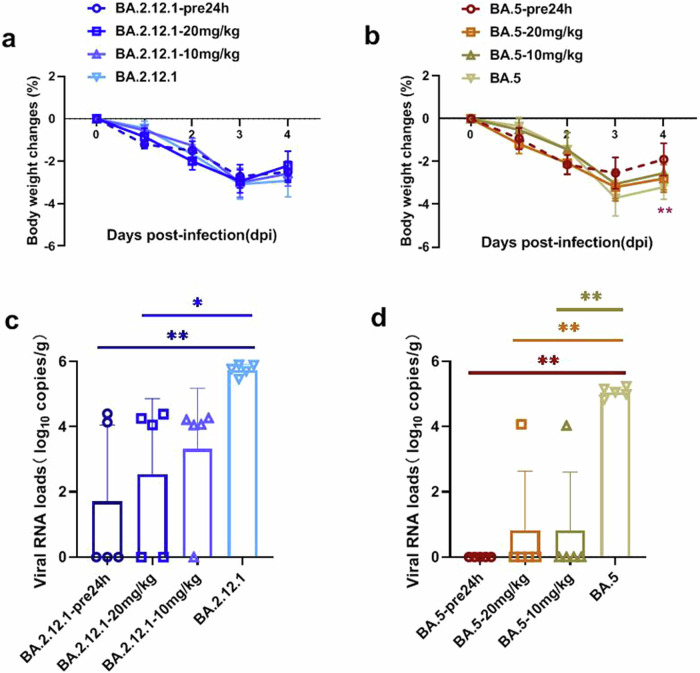
Animal experiments of CR9. hACE2 mice were infected with SARS-CoV-2 stains Omicron BA.2.12.1(**a, c**) or BA.5 (**b, d**). The prevention group were injected with 5 mg/kg purified antibody through the tailvein 24 h before infection, whereas the treatment groups were injected with 10 or 20 mg/kg mAb 2 h after infection. The lungs were collected at 4dpi (*n* = 5) and the viral RNA was measured to analyze the prophylactic or therapeutic efficacy against SARS-CoV-2.The data are represented as mean ±SD. *P < 0.05, **P < 0.01; analyzed using one-way analysis of variance (ANOVA)

To assess the prophylactic potential of CR9, mice were injected intravenously with 5 mg/kg of the antibody 24 h prior to SARS-CoV-2 infection with BA.2.12.1 or BA.5 variants. Therapeutically, mice were administered 10 or 20 mg/kg of CR9 2 h after infection. Control mice received PBS and were infected with the respective variants. Following infection, BA.2.12.1 and BA.5 mice displayed a notable weight loss for three consecutive days, with the prophylactic and therapeutic groups exhibiting a similar weight reduction trend compared to the infected mice(Fig. 3a, b). Notably, the mice pretreated with CR9 against BA.5 recovered significantly from weight loss by the fourth day post-infection, exhibiting a reduced weight loss compared to the BA.5-infected mice (*p* < 0.01) (Fig. 3b). No significant changes were observed in the clinical symptoms or response to external stimuli among the groups. Viral loads were analyzed in the lungs of infected mice. Mice infected with BA.2.12.1 exhibited high viral loads of 10^5.77^ copies/g. In mice pretreated with CR9, lung viral loads decreased significantly by approximately 1.88 log, averaging 10^3.89^ copies/g (*p* < 0.01) at 4 days post-infection (dpi). In the therapeutic groups, mice treated with 20 mg/kg of CR9 showed an average viral load of 10^4.03^ copies/g (*p* < 0.05), while those treated with 10 mg/kg exhibited a load of 10^4.07^ copies/g (*p* > 0.05) (Fig. 3c). Mice infected with BA.5 had lung viral loads averaging 10^5.08^ copies/g. Notably, mice pretreated with CR9 had complete clearance of virus from their lungs (*p* < 0.01). Similarly, 4 out of 5 mice in the 10 and 20 mg/kg therapeutic groups also showed complete virus clearance at 4 dpi (*p* < 0.01) (Fig. 3d).

### Cryo-EM structure of CR9 bound to BA.5

**Fig. 4:**
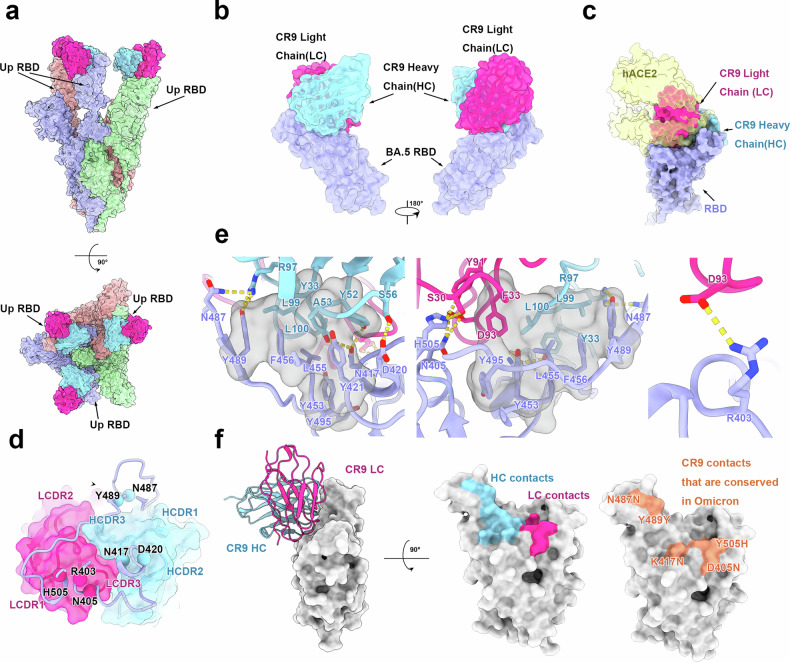
Cryo-EM structures of the spike protein S and RBD complexed with CR9 Fab. **a** Side view and top view of surface representations of the structures of SARS-CoV-2 BA.5 S trimer in complex with Fab CR9 (three up RBDs) with different colors for each S monomer (green, light purple, cyan) and Fab CR9 (light chain: pink; heavy chain: blue). **b** Side views of the binding surface representations of the structures of SARS-CoV-2 BA.5 RBD in complex with Fab CR9. **c** Surface representations of the SARS-CoV-2 BA.5 RBD in complex with Fab CR9 and hACE2(yellow), The hACE2 is presented as surface with 70% transparency. The color scheme is the same as in (**a**). **d** Cartoon representation of the interacting residues in RBD and CDRs in CR9. The residues (R403, N405, N417, D420, N487, Y489 and H505) comprising the epitope are shown as spheres and labeled. The CR9 Fab is presented as surface with 30% transparency and CDRs involved in the interaction with RBD are highlighted. **e** Details of the interactions between CR9 and SARS-CoV-2 BA.5 RBD. Residues involved in the formation of hydrogen bonds are shown as sticks and labeled. Hydrophobic patches are shown in gray surface representation. Hydrogen bonds are depicted as yellow dots. The color scheme is the same as in (**a**). **f** The footprint of CR9 Fabs shown on BA.5 RBD, the conserved sites in Omicron indicated in light salmon

We aimed to understand the structural mechanisms underlying CR9’s broad neutralization activity. Using cryo-electron microscopy, we obtained high-resolution structure of the complex of the pre-fusion-stabilized SARS-CoV-2 BA.5 spike ectodomain trimer and the CR9 Fab fragment. The overall resolution of the asymmetric reconstruction is 3.6 Å. Similar to prior studies, the complex adopts one conformational state with three-up RBD (Fig. 4a). Cryo-EM analysis confirmed full occupancy, with each RBD of the homo-trimeric spike bound by a single Fab. To further enhance the resolution of the binding interface, we determined the structure of the CR9-RBD complex at a resolution of 3.87 Å, providing a robust foundation for detailed interaction analysis (Fig. 4b and Supplementary Figs. S1. S2).

CR9 binds to the apical region of the RBD, partially overlapping the Receptor-Binding Motif (RBM) core (Fig. 4c). The head of the RBD inserted into a cavity constructed by six complementarity-determining regions (CDRs: CDRL1–3 and CDRH1–3), involving extensive hydrophobic and hydrophilic interactions (Fig. 4d). The epitope buried a surface area of ~1100 Å2 and contained seven residues (R403, N405, N417, D420, N487, Y489and H505).At the binding interfaces, CR9 formed a huge hydrophobic pocket with BA.5 RBD formed by Y33, Y52, L99 and L100 from CR9 heavy chain and T421, L455, F456 and Y489 of RBD. Additionally, tight binding is facilitated by14 hydrogen bonds (H bond) and one salt bridges (Fig. 4e).

## Discussion

The emergence of various subtypes of the SARS-CoV-2 virus has sparked a global health crisis due to the severe illness it causes and the large number of deaths it has resulted in refs. ^10,11^ In response to this threat, human monoclonal antibodies (mAbs) have proven to be invaluable in combating emerging infectious diseases like SARS-CoV-2.^12,13^ Studies have shown that neutralizing antibodies have the potential to prevent virus infection,^14^ offering hope for treating novel SARS-CoV-2 strains such as BA.1, BA.2, BA.3, BA.4, and BA.5.^15^ By harnessing specific human antibodies, researchers can gather crucial insights and develop effective antiviral therapies and vaccines to combat SARS-CoV-2 infections.^16,17^ These advancements have the potential to revolutionize the way we approach and manage global health crises in the future.

The study focused on developing Fab phage display libraries from the peripheral B cells of a convalescent individual who had recovered from SARS-CoV-2 infection. By screening the antibody library with purified SARS-CoV-2 S proteins, researchers identified CR9, an effective human neutralizing antibody against SARS-CoV-2. Cryo-electron microscopy of the virion spike protein complexed with Fab fragments unveiled the epitope, created by residues in the RBD. The human monoclonal antibody CR9 specifically targeted the RBD region of the Omicron virus, providing neutralization and preventing the virus from entering cells and causing harm. In vitro studies demonstrated that human mAb CR9 exhibited strong neutralizing activity, effectively inhibiting Omicron replication. This research highlights the potential of CR9 as a promising therapeutic option in combating SARS-CoV-2 infections.

The efficacy of neutralizing monoclonal antibodies (mAbs) in the prevention and treatment of COVID-19 can be categorized into two main groups based on their ability to block the binding of the S proteins to ACE2.^18^ There are currently seven authorized or approved mAbs, including LY-CoV555, LY-CoV016, REGN10987, REGN10933, COV2-2130, COV2-2196, and CT-P59, that function by blocking the binding of the S protein to ACE2. These antibodies are typically used in pairs, targeting epitopes that overlap with the receptor binding motif (RBM), which is known for its structural and evolutionary flexibility. On the other hand, another class of mAbs acts differently by not inhibiting ACE2 binding but neutralizing SARS-CoV-2 through targeting non-RBM epitopes shared by various sarbecoviruses.^19^ According to the previously reported classification based on binding sites,^19^ mAb CR9 belongs to class I antibodies. These antibodies which were most elicited by SARS-CoV-2 early infection, target the receptor binding domain (RBM) and potently neutralize the virus by blocking the interactions between SARS-CoV-2 and hACE2. They are frequently evaded by RBD mutations at the K417, D420, F456, A475and L455 sites. Most earlier class I neutralizing antibodies are already escaped by the B.1.351 (Beta) variant by the K417N mutation, owing to a critical salt-bridge interaction between K417and a negatively charged residue in the antibody. Such as CB6, when RBD K417 is substituted by N417, two crucial salt bridges formed between RBD K417 and D104 of CB6heavy chain, and an additional H bond formed by RBD K417 and CB6 heavy chain Y52 would be abolished, which could lead to a dramatic diminished binding affinity (Supplementary Fig. S3a).In contrast, the footprint of CR9 moved to the locus 489 and 505, which is more conserved in Omicron. Moreover, superposition of WT RBD onto BA.5 RBD-CR9 shows that Y53 of CR9 heavy chain could form one H bond with K417 or N417 meanwhile, which might be connected with the broad neutralization activity of CR9.The CR9 epitope includes 7residues, forming a relatively big patch(Fig. 4f). Among them, 4 are immunological hot binding residues in class I antibodies, including N417, N487, Y489, H505, which indicates a low probability of mutagenesis of the above five loci in Omicron variants (Fig. 4f). In this way, mAb CR9 enables broad spectrum of neutralization capability of neutralizing most Omicron variants. This neutralization mechanism is not same as some other class I broad neutralizing antibodies such as S2E12. The S2E12 paratope recognizes the convex RBM tip, including residues 455 to 458 and 473 to 493 of the SARS-CoV-2 RBD, while site 417 does not participate in the interaction (Supplementary Fig. S3b, c), therefore is not sensitive to mutation K417N. Another identified antibodyBD55-1205^20^ mimics the ACE2 binding mode and its paratope extensively overlap ACE2 interact footprint, and interacts extensively with the main chain of RBD, hence tolerant to all existed mutations including K417N. Compared to these bnAbs, CR9 adopts different broad neutralization strategies.

To probe into the diminished affinity of CR9 to JN.1 RBD, we superposed the JN.1 RBD onto the BA.5-CR9 complex. It turns out that substitution L455S of JN.1 RBD, which is located at the core of CR9-RBDhydrophobicpocket, would abolish the binding activity of CR9 to RBD (Supplementary Fig. S4). The SARS-CoV-2 JN.1-like variants which carry L455S mutation have emerged and rapidly become the dominant variants, and our result partly explain the reason why these variants have escaped a great majority of class I antibodies.

In summary, our study discovered a highly efficient broad-spectrum SARS-CoV-2 neutralizing mAb, which is instructive for future corona virus pandemic prevention. However, the eliminated neutralizing activity to JN.1 tells us that broad neutralizing antibodies appears to be highly time-sensitive, and the evolving landscape may require new strategies. In the future, ACE2 molecular mimicry could provide a high barrier for emergence of escape mutants in spite of the known mutational plasticity of the SARS-CoV-2 RBM, and iterative affinity screening using phage display could be a particularly valuable approach.

The susceptibility of hACE2 mice to SARS-CoV-2 infection prompted us to choose these mice for studying the effectiveness of CR9 as a prophylactic and therapeutic measure.^21^ When groups of infected animals were treated with different doses of CR9, it was observed that viral loads in the lungs were significantly reduced in the prophylactic group, while in the therapeutic group, the reduction was approximately log 1.44 with the higher dose of CR9 for the BA.2.12 strain. Interestingly, complete clearance of viral loads was seen in the prophylactic group for the BA.5 strain, and no viral titer was detected in the lungs of CR9-treated animals in both prophylactic and therapeutic settings for the BA.5 sub-variants. These findings highlight the high efficacy of mAb CR9 against BA.5 infection, making it a promising option for preventing outbreaks and providing treatment for individuals at high risk of exposure to the Omicron variant. This study emphasizes the importance of CR9 in combating the challenges posed by Omicron and its sub-variants, offering hope for effective prevention and control strategies in the future.

In this study, researchers discovered and characterized a monoclonal antibody, known as mAb CR9, which demonstrates significant neutralizing effects against various Omicron sub-variants in laboratory settings. CR9 targets the apical head of the receptor-binding domain (RBD) through interactions with specific regions, inhibiting viral activity effectively. These findings pave the way for the development of potent SARS-CoV-2 vaccines and treatments capable of combating antigenic shifts and potential zoonotic transmissions of sarbecoviruses in the future. The ACE2-blocking properties of mAb CR9 make it a promising candidate for both preventative and therapeutic strategies against the emergence of Omicron and other viral variants, offering hope in the battle against evolving threats.

## Materials and methods

### Ethics statement

The study received ethics review approval (JSJK2023-B002-02) and provided written informed consent. The murine studies were performed in an animal biosafety level 3 (ABSL3) facility using HEPA-filtered isolators, under controlled conditions of temperature (22–24 °C), a relative humidity of 50–60% and 12-h dark/light cycles. Approval for all animal procedures was obtained from the Institutional Animal Care and Use Committee of the Institute of Laboratory Animal Science, Peking Union Medical College (ILAS, PUMC) under the number BLL22011.

### Virus

The SARS-CoV-2 variant Omicron BA.1 (Genbank: OM095411) was discovered at ILAS(PUMC). Other variants of the SARS-CoV-2 virus, including Omicron BA.2.12.1 (GenBank: OP678014), Omicron BA.2 (GenBank: OP678015), Omicron BA.4 (GenBank: OP678017), Omicron BA.5 (GenBank: OP678016), and WH-09 (GenBank: MT093631), were provided by the Guangdong Provincial Center for Disease Control and Prevention (CDC) in China. To confirm the identity of the viral stocks, a plaque-purified viral isolate was expanded as described previously.^22^ The viral concentration in the supernatant was determined using a standard TCID50 assay method to assess the infectivity of the virus.

### Phage library construction

After spending 3-4 weeks recovering in the hospital, peripheral blood mononuclear cells (PBMCs) were extracted from the blood of these resilient patients. The RNA from lymphocytes was carefully isolated and refined using the RNeasy kit from Qiagen, Germany, complying with the manufacturer’s guidelines. Next, cDNA was synthesized by transcribing the total RNA with the Thermo Script reverse transcriptase from Invitrogen, using Oligo-dT as a primer. The heavy and light chain genes were then amplified through polymerase chain reaction (PCR) from the cDNA, and subsequently inserted into the phagemid vector pComb3H obtained from the Scripps Research Institute in the US. Ten clones were randomly selected for sequencing analysis. An anti-SARS-CoV-2 phage antibody library was meticulously created using established primers and techniques.^23^

### Choosing antibodies specific to COVID-19

By utilizing an antibody library, researchers conducted screening with purified SARS-CoV-2 S proteins to isolate potential Fab antibodies. After three rounds of elutriation, the crude Fab antibody preparation underwent testing via an indirect enzyme-linked immunosorbent assay (ELISA), utilizing a 96-well plate coated with purified novel coronavirus S protein at a concentration of 50 ng per well. The second antibody, Horseradish peroxidase (HRP) conjugated anti-human Fab, was employed in the assay. Furthermore, a purification step was conducted using an Anti-Fab affinity chromatography column to isolate human Fab monoclonal antibodies for subsequent characterization and functional analysis. The purity of the human Fab monoclonal antibody was validated through SDS-PAGE analysis.

### IgG1 purification methods

The process of expressing and purifying human IgG1 monoclonal antibody involved constructing a complete human IgG-1 antibody by cloning the variable regions of the heavy chain and light chain of a novel SARS-CoV-2-specific antibody into the PIGG vector. This vector, obtained from Scripps Institute, facilitated the expression of both chains of IgG-1. Through transient transfection of human 293 T cells using Lipofectamine 2000, an IgG1 sample was produced from the PIGG vector. Subsequently, the culture medium containing the antibody was purified using affinity chromatography with a recombinant protein AHiTrap column from GE Healthcare. The purity of the IgG1 was then confirmed through SDS-PAGE and ELISA analysis. This method ensured the production of a high-quality and specific human IgG1 monoclonal antibody for potential therapeutic applications.^24^

### Affinity measurement using Biacore

The study examined the interaction between neutralizing antibodies and the receptor-binding domain (RBD) of SARS-CoV-2 using Surface Plasmon Resonance (SPR) technology. The antibody CR9 was immobilized on a protein A chip and exposed to various concentrations of different SARS-CoV-2 RBD variants. These variants included BA.1, BA.2, BA. 2.12.1, BA.4, and BA.5 at concentrations ranging from 1.25 to 100 nM. By analyzing the binding kinetics and affinity of the antibodies to the RBD variants, researchers aimed to gain insights into the effectiveness of these antibodies in neutralizing the virus. The data obtained was analyzed using BIA Evaluation software to understand the impact of different RBD variants on antibody binding.

### Neutralization assay

The determination of the Inhibitory Concentration 50 (IC_50_) is crucial in the study of antiviral treatments for SARS-CoV-2. This was achieved through observing the cytopathic effects (CPE) of the virus. The process involved serially diluting the antibody two-fold and incubating it with 100 TCID_50_ SARS-CoV-2 at 37 °C, starting with an initial concentration of 50 μg/ml. Each dilution degree was tested in six replicate wells. Subsequently, the mixture was added to Vero-E6 cells in a 96-well-plate and incubated at 37 °C for 3 days. The experiments carried out in triplicate. The IC_50_ value was then calculated by analyzing the initial velocity against various concentrations of the combined molecules with a dose-response curve on GraphPad Prism 8.0 software.

### Pseudovirus production

A novel approach was taken to create a pseudovirus expressing the protein of the novel coronavirus using the HIV-1 backbone. Through the co-transfection of HEK 293 T cells with specific plasmids, including a packaging plasmid psPAX2, a luciferase reporter gene plasmid pLVX-IRES-ZsGreen, and a plasmid pVAX1encoding the SARS-CoV-2 S proteins, a pseudovirus carrying the novel coronavirus protein and expressing luciferase was successfully constructed. This innovative method opens up new possibilities for studying the novel coronavirus and its interactions with host cells.

After incubating cells with the transfection mixture for 24 h, fresh DMEM medium with 10% FBS was added to each Petri dish. The culture medium was then replaced with fresh DMEM medium after overnight incubation. 48 hs post-transfection, the culture supernatant was collected and stored at -80°C for later use.

### Pseudovirus neutralization assay

Pseudovirus neutralization assay measures the ability of antibodies to neutralize pseudoviruses, which are engineered viruses that mimic the structure of real viruses but do not cause illness. This assay is a critical tool in vaccine development and evaluating the efficacy of antibody-based treatments. It allows researchers to assess how well antibodies can prevent viral entry into cells, providing valuable insights into the potential effectiveness of immune responses. Monoclonal antibodies were diluted and tested against pseudovirus in pairs to determine effectiveness. Study shows efficacy of neutralization assay with human ACE2-overexpressed HEK 293 T cells. Pseudoviruses were introduced to 293T-hACE2 cells and incubated at 37 °C for an hour. Subsequently, 293T-hACE2 cells were added to each well and plates were further incubated. After two days, luciferase activity was assessed to determine the effectiveness of the treatment. The IC_50_ value was determined by analyzing the initial velocity and varying concentrations of the compounds in GraphPad Prism 8.0 software. This process allowed for a precise measurement of the inhibitory concentration required to combat the pseudovirus infection in the cells.

### Animal experiments

The study conducted by the Institute of Laboratory Animal Science, Peking Union Medical College utilized specific pathogen-free (SPF) hACE2 mice aged 6-8 weeks to investigate the prophylactic and therapeutic efficacy of purified antibodies against SARS-CoV-2 variants BA.2.12.1 and BA.5. The mice were randomly divided into eight groups, with each group consisting of five mice. For the prophylactic assessment, the mice were injected with 5 mg/kg of the purified antibody 24 h before being intranasally challenged with either SARS-CoV-2 variant at a dosage of 10^5^ TCID_50_ in 50 μL of phosphate-buffered saline (PBS). As for the therapeutic evaluation, mice were first infected with 10^5^ TCID_50_/mL of the SARS-CoV-2 variants and then treated with either 10 or 20 mg/kg of the purified monoclonal antibody after 2 h. Observations were made daily post-infection, recording changes in body weight, clinical symptoms, and responsiveness to external stimuli. The mice were sacrificed at 4 days post-infection, and their lungs were collected for viral load analysis and pathological examination. This research provides valuable insights into the potential effectiveness of purified antibodies in preventing and treating SARS-CoV-2 infections in mice.

### Quantitative RT-PCR for mRNA

Total RNA was extracted using a standard method and then converted to cDNA through RT-PCR.^25^ Subsequently, qRT-PCR was conducted with a specific cycling protocol and primers. The cycling protocol included initial steps at 50 °C for 2 min and 95 °C for 2 min, followed by 40 cycles at 95 °C for 15 seconds and 60 °C for 30 seconds. Final incubation steps were carried out at 95 °C for 15 seconds, 60 °C for 1 minute, and 95 °C for 45 seconds. To detect SARS-CoV-2, specific primers were utilized.

### Protein expression and purification

The plasmid encoding the full spike protein (S protein) of SARS-CoV-2 was utilized as a template to create a variant known as BA.5, which contains multiple mutations. To achieve this, overlapping PCR techniques were employed. The resulting S gene of BA.5 was then inserted into a pCAGGS vector, along with a T4 fibritin trimerization motif, an HRV3C protease site, and a Twin-Strep-tag at the C-terminus, in accordance with previously established methods. Additionally, the receptor-binding domain (RBD) of BA.5 was derived from the S gene using similar PCR techniques and cloned into a pCAGGS vector with a 10His tag. Subsequently, the expression vector containing the modified genes was transiently transfected into HEK293F cells grown in suspension under specific conditions. Following a 72-hour incubation period, the supernatant containing the target proteins was collected, concentrated, and subjected to buffer exchange. The S protein was isolated using chromatography with streptavid in resin and further purified through size exclusion chromatography. Similarly, the RBD protein was purified using nickel-charged resin and size exclusion chromatography, all conducted in buffer solutions of appropriate pH and salt concentration. This meticulous process yielded highly purified BA.5 spike protein and RBD proteins, ready for further characterization and potential applications in research and development. The detailed methodology ensures the integrity and quality of the proteins for future studies exploring the impact of the identified mutations on viral infectivity and immune response.

### Cryo-EM sample collection, data acquisition, and structure determination

Cryo-electron microscopy (Cryo-EM) samples were prepared by combining the BA.5 S trimer with CR9 antibody Fab fragments in a specific ratio and chilling them on ice to form the S-Fab complex. This complex was then carefully placed onto grids that had been treated to enhance adhesion. By applying a brief 6-second blotting at full humidity, the grids were plunged into liquid ethane using a Vitrobot system, ensuring the samples were perfectly preserved. High-quality movies of the complex were captured with a K3 Summit direct detector, with precise defocusing between 1.5–2.7 μm to capture all details. The data acquisition process was automated using Serial EM at a magnification of 22,500x, resulting in an impressive final pixel size of 1.07 Å. This meticulous process allowed for the detailed analysis of the S protein and Fab complex at an atomic level.

In order to enhance the precision of the binding interface between SARS-CoV-2 BA.5 RBD and CR9, researchers successfully determined the structure of BA.5 RBD-CR9. Utilizing the Fab SN1600, a class IV tool known for boosting molecular weight for improved contrast, a cryo-EM sample complex CR9-BA.5 RBD-SN1600 was prepared at a molar ratio of 1:1.2:1.2, resembling the CR9-Spike complex. Data collection took place at 300 kV using an FEI Titan Krios microscope. Movies were recorded with a Gatan K2 Summit direct electron detector by Serial EM, featuring 32 frames lasting 0.2 seconds each and a total dose of 60 e/A°-2s-1. The defocus range fell between 1.5–2.4 μm. The calibrated magnification was set at 22,500×, resulting in a final pixel size of 1.04 A°.

Totally 4541micrographs of the Spike-CR9 complex and 3943 micrographs of RBD-CR9-SN1600were collected. CryoSPARC was used to correct beam-induced motion and average the frames. The defocus value of each micrograph was then estimated using the patch CTF estimation. Then, 2,244,585 Particles of Spike-CR9 complex and 1,118,175 particles of RBD-CR9-SN1600 complex were auto picked and extracted for 2D classification. Next, 364,898good-quality particles of Spike-CR9 complex were primarily selected for Ab-intio reconstruction into 5 classes to generate initial references, and 613,672 particles of RBD-CR9-SN1600 complex were reconstructed in Ab-initio reconstruction in 4classes. Then, heterogenous refinement was performed using the particles and references from Ab-intio reconstruction for both Spike-CR9 and RBD-CR9-SN1600 complexes. At last, 2 classes of Spike-CR9 from heterogenous refinement were selected for homogenous refinement to obtain the final cryo-EM density. One class of RBD-CR9-SN1600 were selected for homogenous refinement and non-uniform refinement for the final cryo-EM density.

Resolutions were assessed using the standard Fourier shell correction at a threshold of 0.143 with ResMap. Data processing steps are detailed in Supplementary Fig. S1 for transparency and reproducibility in evaluating resolution quality.

### Model fitting and refinement

The generation of atom models for the Spike-CR9 complex involved fitting the apo BA.5 spike (PDB:7XNQ) and Fab (PDB:7E5Y) chains into cryo-EM density using Chimera. The resulting structure underwent manual adjustments in Coot, considering protein sequences and density, followed by real-space refinement with Phenix. Similarly, the RBD-CR9-SN1600 complex was derived from the Spike-CR9 complex using Chimera and subsequently refined with Phenix. These refinement procedures are outlined in Supplementary Fig. S1.

### Statistical analysis

Statistical analysis was conducted using GraphPad Prism 8.0 software. Group comparisons were made with a two-tailed, unpaired Student’s *t*-test. Significance levels were set at *p < 0.05 and **p < 0.01 to determine statistical significance.

## Supplementary information

Supplementary Materials

Dataset 1

Dataset 2

## Data Availability

All data are available from the corresponding author on reasonable request.
