# Supplementary material for: A broadly neutralizing antibody against the SARS-CoV-2 Omicron sub-variants BA.1, BA.2, BA.2.12.1, BA.4, and BA.5
Source: Signal Transduct Target Ther. 2025 Jan 13;10:14. doi: 10.1038/s41392-024-02114-6 (PMC11725571; doi:10.1038/s41392-024-02114-6)
Supplement: Supplementary file 1 — Supplementary Materials [file 41392_2024_2114_MOESM1_ESM.docx]

Supplementary Materials for

**A broadly neutralizing antibody against the SARS-CoV-2 Omicron sub-variants BA.1, BA.2, BA.2.12.1, BA.4, and BA.5**

Zhe Chen ^#1^, Leilei Feng ^#2,3^, Lei Wang ^#2,3^, Li Zhang ^#4^, Binyang Zheng ^#4^, Hua Fu ^1^, Fengdi Li^5^, Ligai Liu^6^, Qi Lv^5^, Ran Deng^5^, YanLi Xu^7^, Yongfeng Hu^1^, Jianhua Zheng^1^,Chuan Qin^5^,Linlin Bao*^5^,Xiangxi Wang*^2,3^, and Qi Jin*^1^

Correspondence to: [zdsys@vip.sina.com](mailto:zdsys@vip.sina.com)(Q.J.); xiangxi@ibp.ac.cn (X.W.); [bllmsl@aliyun.com(L.B.)](mailto:bllmsl@aliyun.com(L.B.))

This PDF file includes:

Figures. S1 to S4


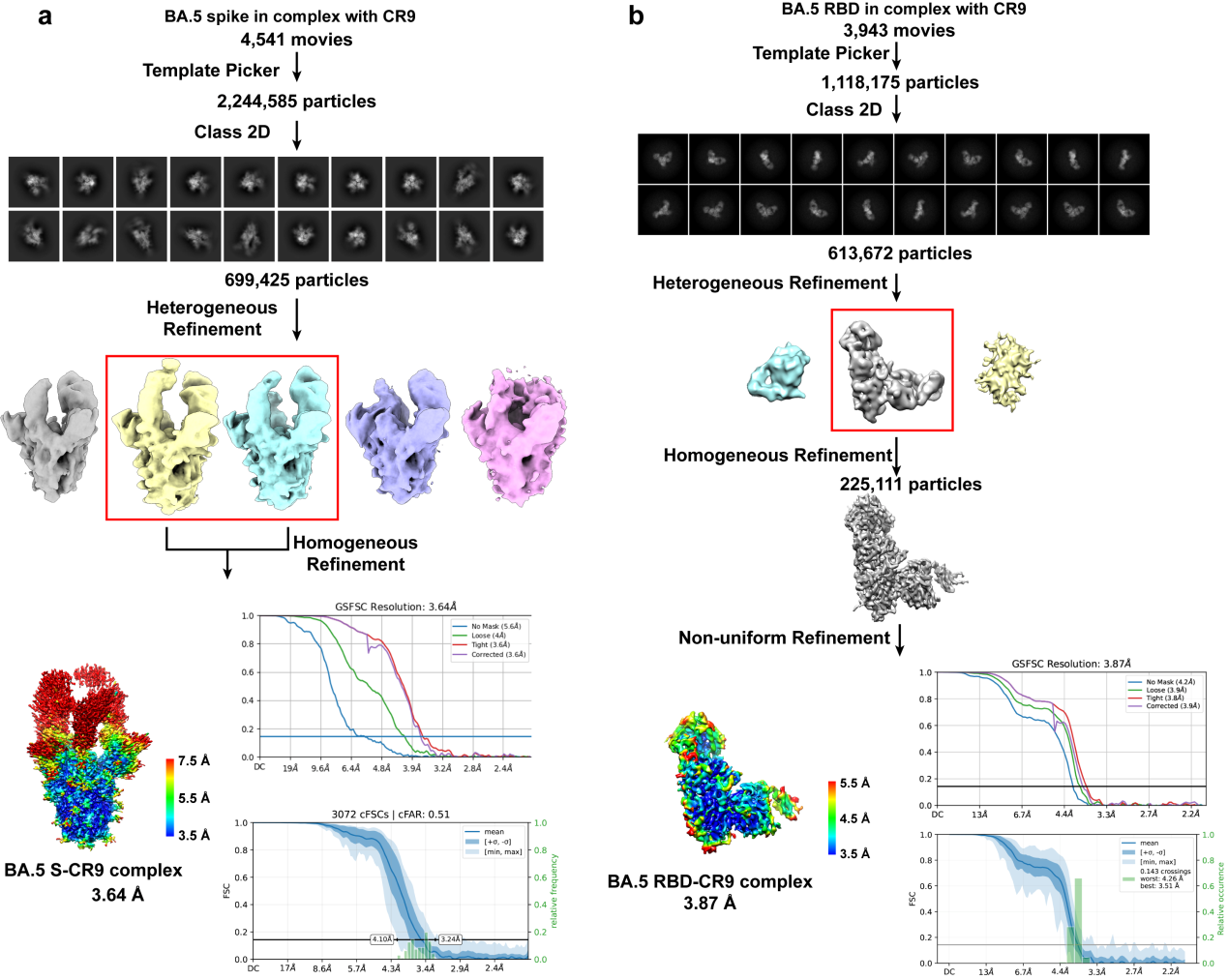


Figures. S1

Flowcharts for structure determinations of (**a**) BA.5 S-CR9 complex, (**b**) BA.5 RBD-CR9 complex.


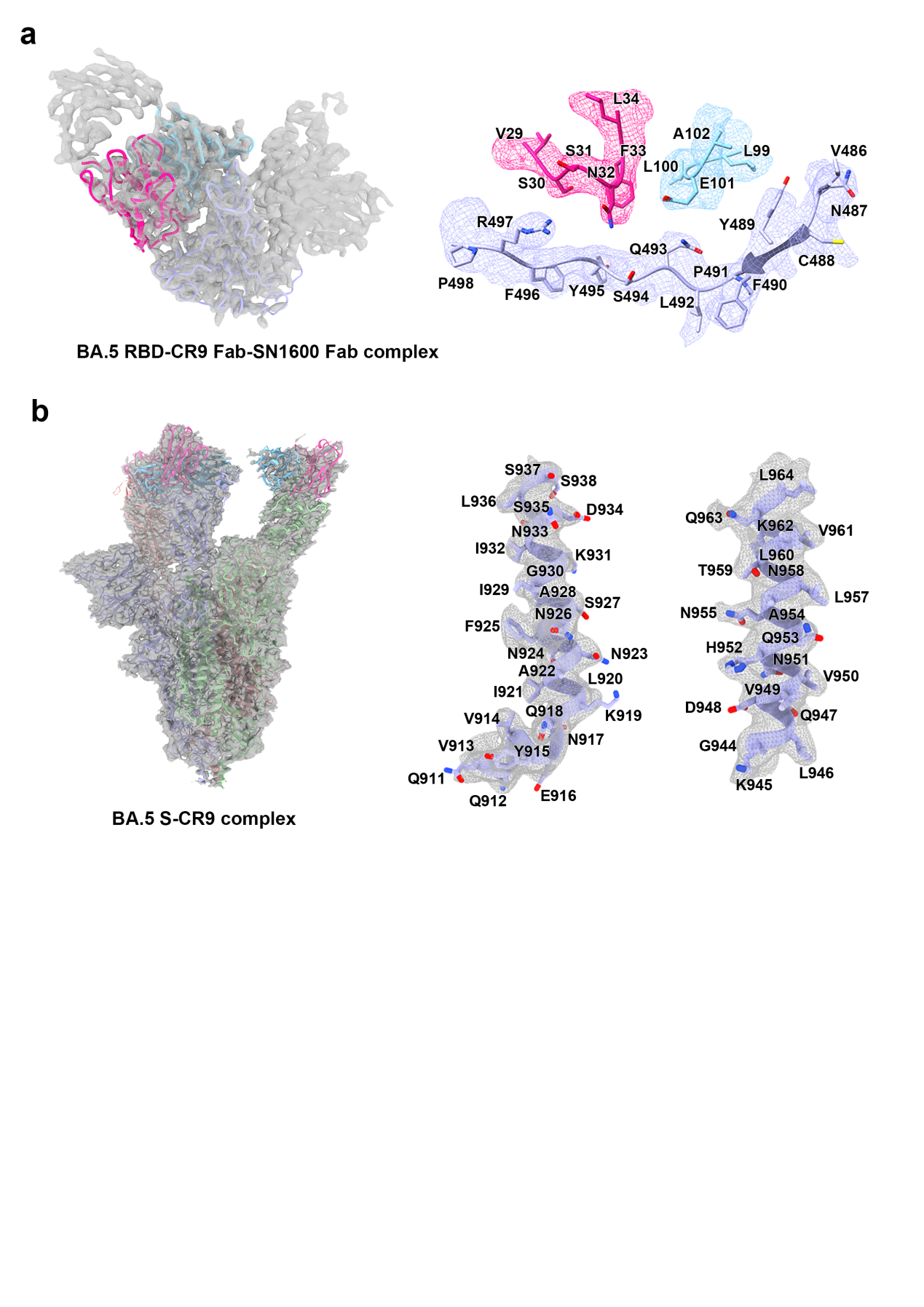


Figures. S2

Cryo-EM and local map of the binding interface of BA.5 RBD-CR9 complex(**a**), cryo-EM and local map of the S-CR9 complex(**b**). Residues are shown as sticks with oxygen colored in red, nitrogen colored in blue and sulfurs colored in yellow，different colors for RBD (cyan), and Fab CR9 (light chain: pink; heavy chain: blue) were shown. The electron density map shown here were generated in chimeraX-1.5 shown in mesh style with a contour level of RBD-CR9 complex 0.105 and S-CR9 complex 0.401.


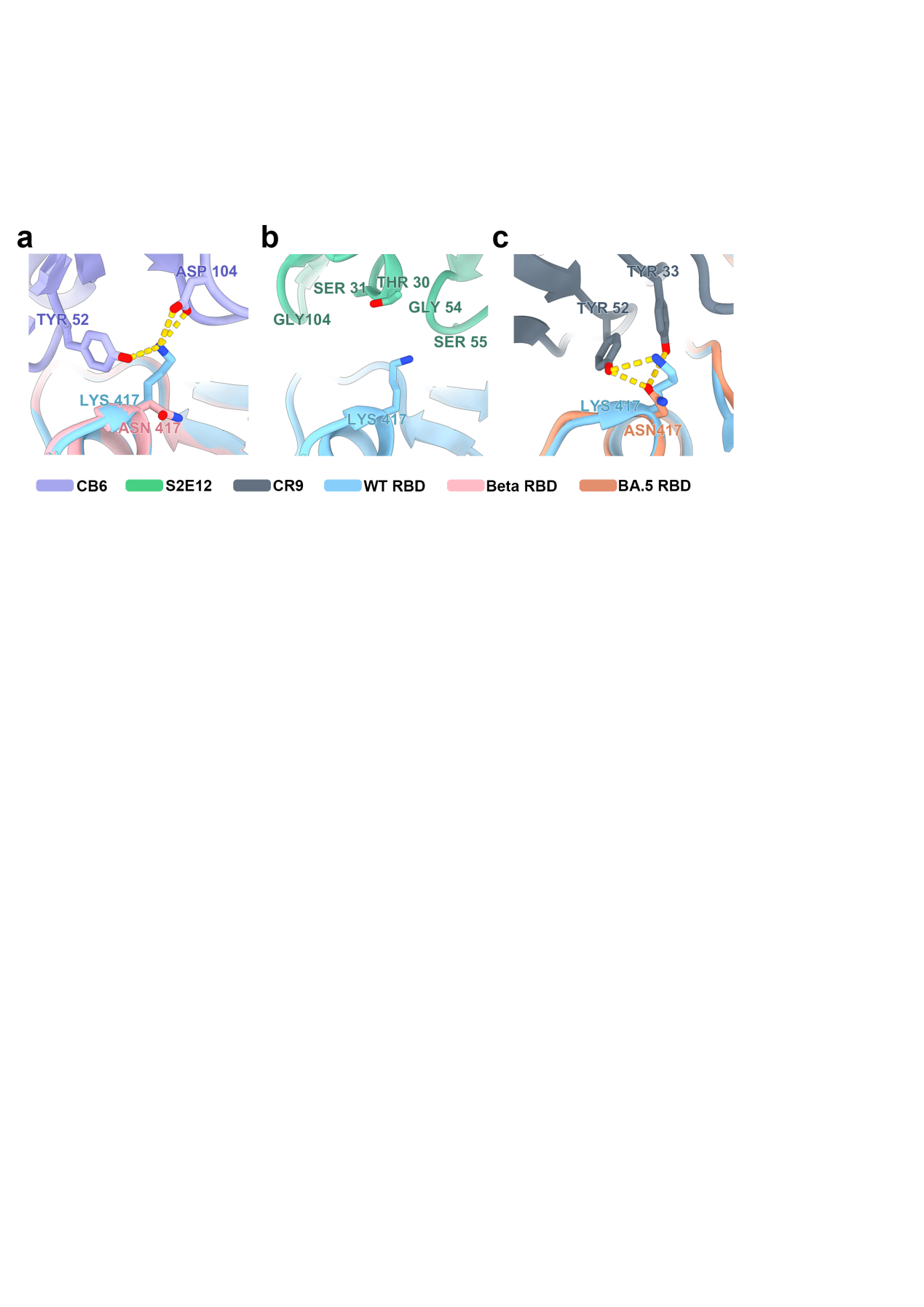


Fig. S3

Comparison of RBD-Antibody binding interface around site K/N 417. (**a**) Alignment of Beta RBD and CB6-WT RBD complex (PDB: 7XEI)around site K/N 417, (**b**) Interactions of S2E12 and WT RBD around site K417 (PDB: 7K45) and (**c**) Alignment of WT RBD and CR9-BA.5 RBD around site K/N 417.


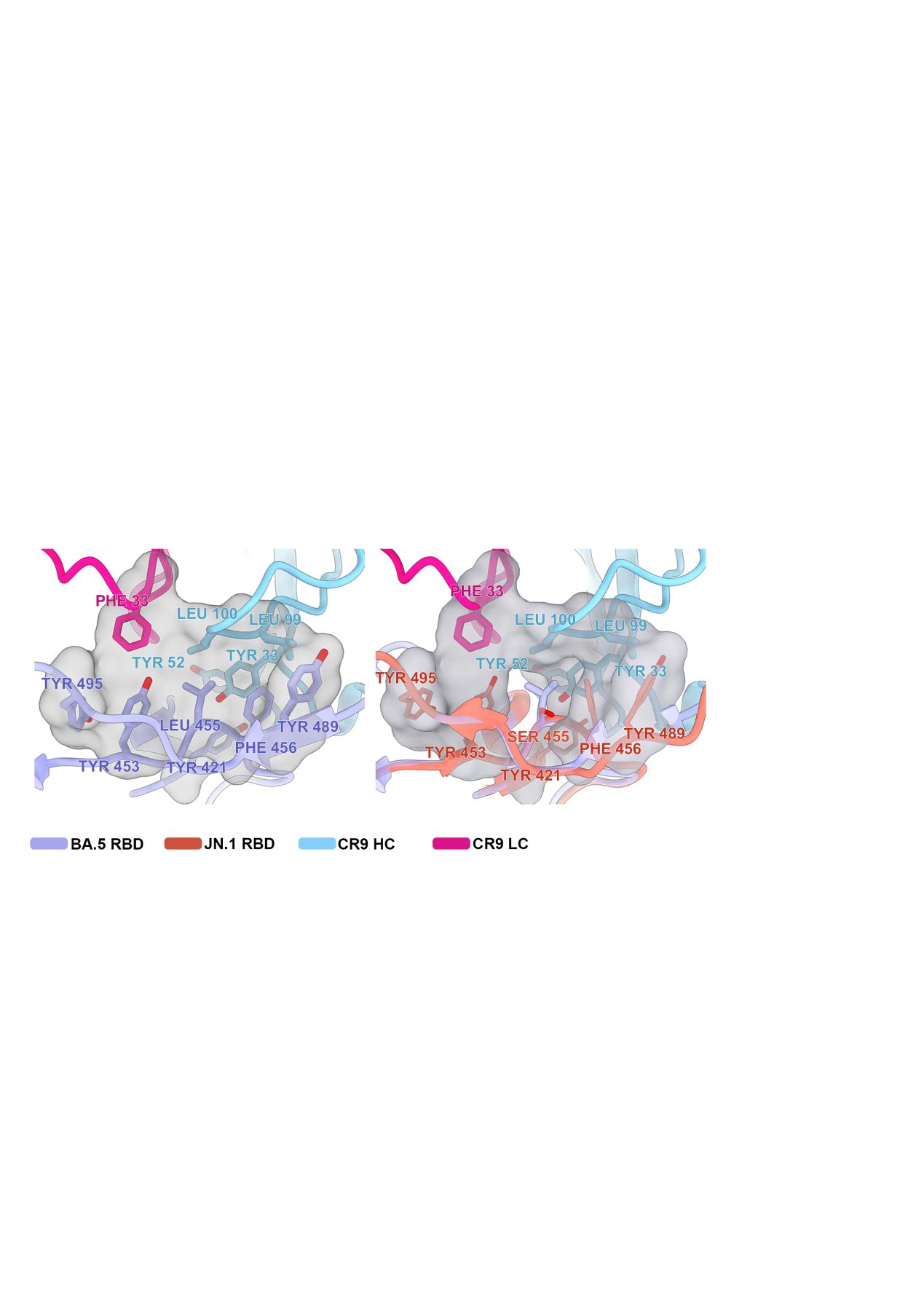


Fig. S4

Hydrophobic interactions comparation of JN.1 RBD (PDB:8Y18) intoBA.5 RBD- CR9 complex.
