# Supplementary material for: A broadly neutralizing antibody against the SARS-CoV-2 Omicron sub-variants BA.1, BA.2, BA.2.12.1, BA.4, and BA.5
Source: Signal Transduct Target Ther. 2025 Jan 13;10:14. doi: 10.1038/s41392-024-02114-6 (PMC11725571; doi:10.1038/s41392-024-02114-6)
Supplement: Supplementary file 2 — Dataset 1 [file 41392_2024_2114_MOESM2_ESM.pdf]

Full wwPDB EM Validation Report ⓘ

Feb 1, 2024 – 06:27 PM JST

PDB ID	:	8XSD
EMDB ID	:	EMD-38616
Title	:	BA.5 RBD in complex with CR9
Deposited on	:	2024-01-09
Resolution	:	3.55 Å(reported)

This wwPDB validation report is for manuscript review

This is a Full wwPDB EM Validation Report.

This report is produced by the wwPDB biocuration pipeline after annotation of the structure.

We welcome your comments at *validation@mail.wwpdb.org*
A user guide is available at
https://www.wwpdb.org/validation/2017/EMValidationReportHelp
with specific help available everywhere you see the ⓘ symbol.

The types of validation reports are described at
http://www.wwpdb.org/validation/2017/FAQs#types.

For Manuscript Review

The following versions of software and data (see references ⓘ) were used in the production of this report:

EMDB validation analysis	:	0.0.1.dev70
MolProbity	:	4.02b-467
Percentile statistics	:	20191225.v01 (using entries in the PDB archive December 25th 2019)
MapQ	:	1.9.9
Ideal geometry (proteins)	:	Engh & Huber (2001)
Ideal geometry (DNA, RNA)	:	Parkinson et al. (1996)
Validation Pipeline (wwPDB-VP)	:	2.36

1 Overall quality at a glance (i)

The following experimental techniques were used to determine the structure:
ELECTRON MICROSCOPY

The reported resolution of this entry is 3.55 Å.

Percentile scores (ranging between 0-100) for global validation metrics of the entry are shown in the following graphic. The table shows the number of entries on which the scores are based.

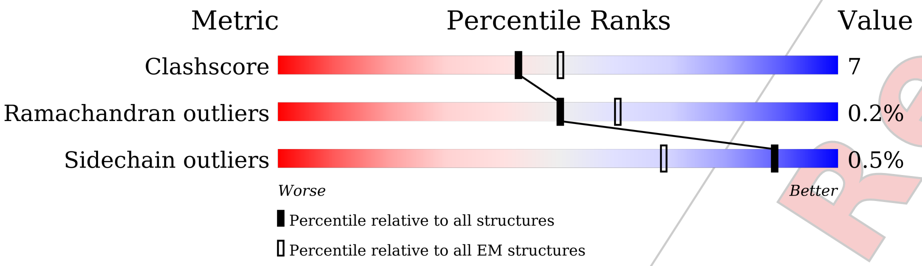

Metric	Whole archive (#Entries)	EM structures (#Entries)
Clashscore	158937	4297
Ramachandran outliers	154571	4023
Sidechain outliers	154315	3826

The table below summarises the geometric issues observed across the polymeric chains and their fit to the map. The red, orange, yellow and green segments of the bar indicate the fraction of residues that contain outliers for >=3, 2, 1 and 0 types of geometric quality criteria respectively. A grey segment represents the fraction of residues that are not modelled. The numeric value for each fraction is indicated below the corresponding segment, with a dot representing fractions <=5% The upper red bar (where present) indicates the fraction of residues that have poor fit to the EM map (all-atom inclusion < 40%). The numeric value is given above the bar.

Mol	Chain	Length	Quality of chain
1	A	1289	58% / 67% 13% • 19%
1	B	1289	69% / 70% 11% 18%
1	C	1289	58% / 71% 11% 19%
2	G	115	100% / 70% 30%
2	H	115	91% / 70% 30%
2	I	115	97% / 74% 26%
3	J	107	100% / 71% 28% •
3	K	107	99% / 77% 22% •

Continued on next page...

For Manuscript Review

Continued from previous page...

Mol	Chain	Length	Quality of chain
3	L	107	96% / 78% 21% •

For Manuscript Review

2 Entry composition (i)

There are 3 unique types of molecules in this entry. The entry contains 29708 atoms, of which 0 are hydrogens and 0 are deuteriums.

In the tables below, the AltConf column contains the number of residues with at least one atom in alternate conformation and the Trace column contains the number of residues modelled with at most 2 atoms.

- Molecule 1 is a protein called Spike glycoprotein.

Mol	Chain	Residues	Atoms					AltConf	Trace
1	A	1048	Total 8201	C 5249	N 1363	O 1551	S 38	0	0
1	B	1055	Total 8255	C 5280	N 1373	O 1564	S 38	0	0
1	C	1050	Total 8215	C 5254	N 1367	O 1557	S 37	0	0

There are 372 discrepancies between the modelled and reference sequences:

Chain	Residue	Modelled	Actual	Comment	Reference
A	22	ILE	THR	variant	UNP P0DTC2
A	?	-	LEU	deletion	UNP P0DTC2
A	?	-	PRO	deletion	UNP P0DTC2
A	?	-	PRO	deletion	UNP P0DTC2
A	27	SER	ALA	variant	UNP P0DTC2
A	?	-	HIS	deletion	UNP P0DTC2
A	?	-	VAL	deletion	UNP P0DTC2
A	140	ASP	GLY	variant	UNP P0DTC2
A	211	GLY	VAL	variant	UNP P0DTC2
A	337	ASP	GLY	variant	UNP P0DTC2
A	369	PHE	SER	variant	UNP P0DTC2
A	371	PRO	SER	variant	UNP P0DTC2
A	373	PHE	SER	variant	UNP P0DTC2
A	374	ALA	THR	variant	UNP P0DTC2
A	403	ASN	ASP	variant	UNP P0DTC2
A	406	SER	ARG	variant	UNP P0DTC2
A	415	ASN	LYS	variant	UNP P0DTC2
A	438	LYS	ASN	variant	UNP P0DTC2
A	450	ARG	LEU	variant	UNP P0DTC2
A	475	ASN	SER	variant	UNP P0DTC2
A	476	LYS	THR	variant	UNP P0DTC2
A	482	ALA	GLU	variant	UNP P0DTC2
A	484	VAL	PHE	variant	UNP P0DTC2
A	496	ARG	GLN	variant	UNP P0DTC2

Continued on next page...

For Manuscript Review

Continued from previous page...

Chain	Residue	Modelled	Actual	Comment	Reference
A	499	TYR	ASN	variant	UNP P0DTC2
A	503	HIS	TYR	variant	UNP P0DTC2
A	612	GLY	ASP	variant	UNP P0DTC2
A	653	TYR	HIS	variant	UNP P0DTC2
A	656	SER	ASN	variant	UNP P0DTC2
A	677	LYS	ASN	variant	UNP P0DTC2
A	679	HIS	PRO	variant	UNP P0DTC2
A	681	ALA	ARG	variant	UNP P0DTC2
A	683	ALA	ARG	variant	UNP P0DTC2
A	762	LYS	ASN	variant	UNP P0DTC2
A	794	TYR	ASP	variant	UNP P0DTC2
A	815	PRO	PHE	variant	UNP P0DTC2
A	890	PRO	ALA	variant	UNP P0DTC2
A	897	PRO	ALA	variant	UNP P0DTC2
A	940	PRO	ALA	variant	UNP P0DTC2
A	952	HIS	GLN	variant	UNP P0DTC2
A	967	LYS	ASN	variant	UNP P0DTC2
A	984	PRO	LYS	variant	UNP P0DTC2
A	985	PRO	VAL	variant	UNP P0DTC2
A	1212	GLY	-	expression tag	UNP P0DTC2
A	1213	GLY	-	expression tag	UNP P0DTC2
A	1214	SER	-	expression tag	UNP P0DTC2
A	1215	GLY	-	expression tag	UNP P0DTC2
A	1216	GLY	-	expression tag	UNP P0DTC2
A	1217	SER	-	expression tag	UNP P0DTC2
A	1218	TYR	-	expression tag	UNP P0DTC2
A	1219	ILE	-	expression tag	UNP P0DTC2
A	1220	PRO	-	expression tag	UNP P0DTC2
A	1221	GLU	-	expression tag	UNP P0DTC2
A	1222	ALA	-	expression tag	UNP P0DTC2
A	1223	PRO	-	expression tag	UNP P0DTC2
A	1224	ARG	-	expression tag	UNP P0DTC2
A	1225	ASP	-	expression tag	UNP P0DTC2
A	1226	GLY	-	expression tag	UNP P0DTC2
A	1227	GLN	-	expression tag	UNP P0DTC2
A	1228	ALA	-	expression tag	UNP P0DTC2
A	1229	TYR	-	expression tag	UNP P0DTC2
A	1230	VAL	-	expression tag	UNP P0DTC2
A	1231	ARG	-	expression tag	UNP P0DTC2
A	1232	LYS	-	expression tag	UNP P0DTC2
A	1233	ASP	-	expression tag	UNP P0DTC2
A	1234	GLY	-	expression tag	UNP P0DTC2

Continued on next page...

For Manuscript Review

Continued from previous page...

Chain	Residue	Modelled	Actual	Comment	Reference
A	1235	GLU	-	expression tag	UNP P0DTC2
A	1236	TRP	-	expression tag	UNP P0DTC2
A	1237	VAL	-	expression tag	UNP P0DTC2
A	1238	LEU	-	expression tag	UNP P0DTC2
A	1239	LEU	-	expression tag	UNP P0DTC2
A	1240	SER	-	expression tag	UNP P0DTC2
A	1241	THR	-	expression tag	UNP P0DTC2
A	1242	PHE	-	expression tag	UNP P0DTC2
A	1243	LEU	-	expression tag	UNP P0DTC2
A	1244	GLY	-	expression tag	UNP P0DTC2
A	1245	ARG	-	expression tag	UNP P0DTC2
A	1246	SER	-	expression tag	UNP P0DTC2
A	1247	LEU	-	expression tag	UNP P0DTC2
A	1248	GLU	-	expression tag	UNP P0DTC2
A	1249	VAL	-	expression tag	UNP P0DTC2
A	1250	LEU	-	expression tag	UNP P0DTC2
A	1251	PHE	-	expression tag	UNP P0DTC2
A	1252	GLN	-	expression tag	UNP P0DTC2
A	1253	GLY	-	expression tag	UNP P0DTC2
A	1254	PRO	-	expression tag	UNP P0DTC2
A	1255	GLY	-	expression tag	UNP P0DTC2
A	1256	TRP	-	expression tag	UNP P0DTC2
A	1257	SER	-	expression tag	UNP P0DTC2
A	1258	HIS	-	expression tag	UNP P0DTC2
A	1259	PRO	-	expression tag	UNP P0DTC2
A	1260	GLN	-	expression tag	UNP P0DTC2
A	1261	PHE	-	expression tag	UNP P0DTC2
A	1262	GLU	-	expression tag	UNP P0DTC2
A	1263	LYS	-	expression tag	UNP P0DTC2
A	1264	GLY	-	expression tag	UNP P0DTC2
A	1265	GLY	-	expression tag	UNP P0DTC2
A	1266	GLY	-	expression tag	UNP P0DTC2
A	1267	SER	-	expression tag	UNP P0DTC2
A	1268	GLY	-	expression tag	UNP P0DTC2
A	1269	GLY	-	expression tag	UNP P0DTC2
A	1270	GLY	-	expression tag	UNP P0DTC2
A	1271	SER	-	expression tag	UNP P0DTC2
A	1272	GLY	-	expression tag	UNP P0DTC2
A	1273	GLY	-	expression tag	UNP P0DTC2
A	1274	SER	-	expression tag	UNP P0DTC2
A	1275	SER	-	expression tag	UNP P0DTC2
A	1276	ALA	-	expression tag	UNP P0DTC2

Continued on next page...

For Manuscript Review

Continued from previous page...

Chain	Residue	Modelled	Actual	Comment	Reference
A	1277	TRP	-	expression tag	UNP P0DTC2
A	1278	SER	-	expression tag	UNP P0DTC2
A	1279	HIS	-	expression tag	UNP P0DTC2
A	1280	PRO	-	expression tag	UNP P0DTC2
A	1281	GLN	-	expression tag	UNP P0DTC2
A	1282	PHE	-	expression tag	UNP P0DTC2
A	1283	GLU	-	expression tag	UNP P0DTC2
A	1284	LYS	-	expression tag	UNP P0DTC2
A	1285	HIS	-	expression tag	UNP P0DTC2
A	1286	HIS	-	expression tag	UNP P0DTC2
A	1287	HIS	-	expression tag	UNP P0DTC2
A	1288	HIS	-	expression tag	UNP P0DTC2
A	1289	HIS	-	expression tag	UNP P0DTC2
A	1290	HIS	-	expression tag	UNP P0DTC2
A	1291	HIS	-	expression tag	UNP P0DTC2
A	1292	HIS	-	expression tag	UNP P0DTC2
B	22	ILE	THR	variant	UNP P0DTC2
B	?	-	LEU	deletion	UNP P0DTC2
B	?	-	PRO	deletion	UNP P0DTC2
B	?	-	PRO	deletion	UNP P0DTC2
B	27	SER	ALA	variant	UNP P0DTC2
B	?	-	HIS	deletion	UNP P0DTC2
B	?	-	VAL	deletion	UNP P0DTC2
B	140	ASP	GLY	variant	UNP P0DTC2
B	211	GLY	VAL	variant	UNP P0DTC2
B	337	ASP	GLY	variant	UNP P0DTC2
B	369	PHE	SER	variant	UNP P0DTC2
B	371	PRO	SER	variant	UNP P0DTC2
B	373	PHE	SER	variant	UNP P0DTC2
B	374	ALA	THR	variant	UNP P0DTC2
B	403	ASN	ASP	variant	UNP P0DTC2
B	406	SER	ARG	variant	UNP P0DTC2
B	415	ASN	LYS	variant	UNP P0DTC2
B	438	LYS	ASN	variant	UNP P0DTC2
B	450	ARG	LEU	variant	UNP P0DTC2
B	475	ASN	SER	variant	UNP P0DTC2
B	476	LYS	THR	variant	UNP P0DTC2
B	482	ALA	GLU	variant	UNP P0DTC2
B	484	VAL	PHE	variant	UNP P0DTC2
B	496	ARG	GLN	variant	UNP P0DTC2
B	499	TYR	ASN	variant	UNP P0DTC2
B	503	HIS	TYR	variant	UNP P0DTC2

Continued on next page...

For Manuscript Review

Continued from previous page...

Chain	Residue	Modelled	Actual	Comment	Reference
B	612	GLY	ASP	variant	UNP P0DTC2
B	653	TYR	HIS	variant	UNP P0DTC2
B	656	SER	ASN	variant	UNP P0DTC2
B	677	LYS	ASN	variant	UNP P0DTC2
B	679	HIS	PRO	variant	UNP P0DTC2
B	681	ALA	ARG	variant	UNP P0DTC2
B	683	ALA	ARG	variant	UNP P0DTC2
B	762	LYS	ASN	variant	UNP P0DTC2
B	794	TYR	ASP	variant	UNP P0DTC2
B	815	PRO	PHE	variant	UNP P0DTC2
B	890	PRO	ALA	variant	UNP P0DTC2
B	897	PRO	ALA	variant	UNP P0DTC2
B	940	PRO	ALA	variant	UNP P0DTC2
B	952	HIS	GLN	variant	UNP P0DTC2
B	967	LYS	ASN	variant	UNP P0DTC2
B	984	PRO	LYS	variant	UNP P0DTC2
B	985	PRO	VAL	variant	UNP P0DTC2
B	1212	GLY	-	expression tag	UNP P0DTC2
B	1213	GLY	-	expression tag	UNP P0DTC2
B	1214	SER	-	expression tag	UNP P0DTC2
B	1215	GLY	-	expression tag	UNP P0DTC2
B	1216	GLY	-	expression tag	UNP P0DTC2
B	1217	SER	-	expression tag	UNP P0DTC2
B	1218	TYR	-	expression tag	UNP P0DTC2
B	1219	ILE	-	expression tag	UNP P0DTC2
B	1220	PRO	-	expression tag	UNP P0DTC2
B	1221	GLU	-	expression tag	UNP P0DTC2
B	1222	ALA	-	expression tag	UNP P0DTC2
B	1223	PRO	-	expression tag	UNP P0DTC2
B	1224	ARG	-	expression tag	UNP P0DTC2
B	1225	ASP	-	expression tag	UNP P0DTC2
B	1226	GLY	-	expression tag	UNP P0DTC2
B	1227	GLN	-	expression tag	UNP P0DTC2
B	1228	ALA	-	expression tag	UNP P0DTC2
B	1229	TYR	-	expression tag	UNP P0DTC2
B	1230	VAL	-	expression tag	UNP P0DTC2
B	1231	ARG	-	expression tag	UNP P0DTC2
B	1232	LYS	-	expression tag	UNP P0DTC2
B	1233	ASP	-	expression tag	UNP P0DTC2
B	1234	GLY	-	expression tag	UNP P0DTC2
B	1235	GLU	-	expression tag	UNP P0DTC2
B	1236	TRP	-	expression tag	UNP P0DTC2

Continued on next page...

For Manuscript Review

Continued from previous page...

Chain	Residue	Modelled	Actual	Comment	Reference
B	1237	VAL	-	expression tag	UNP P0DTC2
B	1238	LEU	-	expression tag	UNP P0DTC2
B	1239	LEU	-	expression tag	UNP P0DTC2
B	1240	SER	-	expression tag	UNP P0DTC2
B	1241	THR	-	expression tag	UNP P0DTC2
B	1242	PHE	-	expression tag	UNP P0DTC2
B	1243	LEU	-	expression tag	UNP P0DTC2
B	1244	GLY	-	expression tag	UNP P0DTC2
B	1245	ARG	-	expression tag	UNP P0DTC2
B	1246	SER	-	expression tag	UNP P0DTC2
B	1247	LEU	-	expression tag	UNP P0DTC2
B	1248	GLU	-	expression tag	UNP P0DTC2
B	1249	VAL	-	expression tag	UNP P0DTC2
B	1250	LEU	-	expression tag	UNP P0DTC2
B	1251	PHE	-	expression tag	UNP P0DTC2
B	1252	GLN	-	expression tag	UNP P0DTC2
B	1253	GLY	-	expression tag	UNP P0DTC2
B	1254	PRO	-	expression tag	UNP P0DTC2
B	1255	GLY	-	expression tag	UNP P0DTC2
B	1256	TRP	-	expression tag	UNP P0DTC2
B	1257	SER	-	expression tag	UNP P0DTC2
B	1258	HIS	-	expression tag	UNP P0DTC2
B	1259	PRO	-	expression tag	UNP P0DTC2
B	1260	GLN	-	expression tag	UNP P0DTC2
B	1261	PHE	-	expression tag	UNP P0DTC2
B	1262	GLU	-	expression tag	UNP P0DTC2
B	1263	LYS	-	expression tag	UNP P0DTC2
B	1264	GLY	-	expression tag	UNP P0DTC2
B	1265	GLY	-	expression tag	UNP P0DTC2
B	1266	GLY	-	expression tag	UNP P0DTC2
B	1267	SER	-	expression tag	UNP P0DTC2
B	1268	GLY	-	expression tag	UNP P0DTC2
B	1269	GLY	-	expression tag	UNP P0DTC2
B	1270	GLY	-	expression tag	UNP P0DTC2
B	1271	SER	-	expression tag	UNP P0DTC2
B	1272	GLY	-	expression tag	UNP P0DTC2
B	1273	GLY	-	expression tag	UNP P0DTC2
B	1274	SER	-	expression tag	UNP P0DTC2
B	1275	SER	-	expression tag	UNP P0DTC2
B	1276	ALA	-	expression tag	UNP P0DTC2
B	1277	TRP	-	expression tag	UNP P0DTC2
B	1278	SER	-	expression tag	UNP P0DTC2

Continued on next page...

For Manuscript Review

Continued from previous page...

Chain	Residue	Modelled	Actual	Comment	Reference
B	1279	HIS	-	expression tag	UNP P0DTC2
B	1280	PRO	-	expression tag	UNP P0DTC2
B	1281	GLN	-	expression tag	UNP P0DTC2
B	1282	PHE	-	expression tag	UNP P0DTC2
B	1283	GLU	-	expression tag	UNP P0DTC2
B	1284	LYS	-	expression tag	UNP P0DTC2
B	1285	HIS	-	expression tag	UNP P0DTC2
B	1286	HIS	-	expression tag	UNP P0DTC2
B	1287	HIS	-	expression tag	UNP P0DTC2
B	1288	HIS	-	expression tag	UNP P0DTC2
B	1289	HIS	-	expression tag	UNP P0DTC2
B	1290	HIS	-	expression tag	UNP P0DTC2
B	1291	HIS	-	expression tag	UNP P0DTC2
B	1292	HIS	-	expression tag	UNP P0DTC2
C	22	ILE	THR	variant	UNP P0DTC2
C	?	-	LEU	deletion	UNP P0DTC2
C	?	-	PRO	deletion	UNP P0DTC2
C	?	-	PRO	deletion	UNP P0DTC2
C	27	SER	ALA	variant	UNP P0DTC2
C	?	-	HIS	deletion	UNP P0DTC2
C	?	-	VAL	deletion	UNP P0DTC2
C	140	ASP	GLY	variant	UNP P0DTC2
C	211	GLY	VAL	variant	UNP P0DTC2
C	337	ASP	GLY	variant	UNP P0DTC2
C	369	PHE	SER	variant	UNP P0DTC2
C	371	PRO	SER	variant	UNP P0DTC2
C	373	PHE	SER	variant	UNP P0DTC2
C	374	ALA	THR	variant	UNP P0DTC2
C	403	ASN	ASP	variant	UNP P0DTC2
C	406	SER	ARG	variant	UNP P0DTC2
C	415	ASN	LYS	variant	UNP P0DTC2
C	438	LYS	ASN	variant	UNP P0DTC2
C	450	ARG	LEU	variant	UNP P0DTC2
C	475	ASN	SER	variant	UNP P0DTC2
C	476	LYS	THR	variant	UNP P0DTC2
C	482	ALA	GLU	variant	UNP P0DTC2
C	484	VAL	PHE	variant	UNP P0DTC2
C	496	ARG	GLN	variant	UNP P0DTC2
C	499	TYR	ASN	variant	UNP P0DTC2
C	503	HIS	TYR	variant	UNP P0DTC2
C	612	GLY	ASP	variant	UNP P0DTC2
C	653	TYR	HIS	variant	UNP P0DTC2

Continued on next page...

For Manuscript Review

Continued from previous page...

Chain	Residue	Modelled	Actual	Comment	Reference
C	656	SER	ASN	variant	UNP P0DTC2
C	677	LYS	ASN	variant	UNP P0DTC2
C	679	HIS	PRO	variant	UNP P0DTC2
C	681	ALA	ARG	variant	UNP P0DTC2
C	683	ALA	ARG	variant	UNP P0DTC2
C	762	LYS	ASN	variant	UNP P0DTC2
C	794	TYR	ASP	variant	UNP P0DTC2
C	815	PRO	PHE	variant	UNP P0DTC2
C	890	PRO	ALA	variant	UNP P0DTC2
C	897	PRO	ALA	variant	UNP P0DTC2
C	940	PRO	ALA	variant	UNP P0DTC2
C	952	HIS	GLN	variant	UNP P0DTC2
C	967	LYS	ASN	variant	UNP P0DTC2
C	984	PRO	LYS	variant	UNP P0DTC2
C	985	PRO	VAL	variant	UNP P0DTC2
C	1212	GLY	-	expression tag	UNP P0DTC2
C	1213	GLY	-	expression tag	UNP P0DTC2
C	1214	SER	-	expression tag	UNP P0DTC2
C	1215	GLY	-	expression tag	UNP P0DTC2
C	1216	GLY	-	expression tag	UNP P0DTC2
C	1217	SER	-	expression tag	UNP P0DTC2
C	1218	TYR	-	expression tag	UNP P0DTC2
C	1219	ILE	-	expression tag	UNP P0DTC2
C	1220	PRO	-	expression tag	UNP P0DTC2
C	1221	GLU	-	expression tag	UNP P0DTC2
C	1222	ALA	-	expression tag	UNP P0DTC2
C	1223	PRO	-	expression tag	UNP P0DTC2
C	1224	ARG	-	expression tag	UNP P0DTC2
C	1225	ASP	-	expression tag	UNP P0DTC2
C	1226	GLY	-	expression tag	UNP P0DTC2
C	1227	GLN	-	expression tag	UNP P0DTC2
C	1228	ALA	-	expression tag	UNP P0DTC2
C	1229	TYR	-	expression tag	UNP P0DTC2
C	1230	VAL	-	expression tag	UNP P0DTC2
C	1231	ARG	-	expression tag	UNP P0DTC2
C	1232	LYS	-	expression tag	UNP P0DTC2
C	1233	ASP	-	expression tag	UNP P0DTC2
C	1234	GLY	-	expression tag	UNP P0DTC2
C	1235	GLU	-	expression tag	UNP P0DTC2
C	1236	TRP	-	expression tag	UNP P0DTC2
C	1237	VAL	-	expression tag	UNP P0DTC2
C	1238	LEU	-	expression tag	UNP P0DTC2

Continued on next page...

For Manuscript Review

Continued from previous page...

Chain	Residue	Modelled	Actual	Comment	Reference
C	1239	LEU	-	expression tag	UNP P0DTC2
C	1240	SER	-	expression tag	UNP P0DTC2
C	1241	THR	-	expression tag	UNP P0DTC2
C	1242	PHE	-	expression tag	UNP P0DTC2
C	1243	LEU	-	expression tag	UNP P0DTC2
C	1244	GLY	-	expression tag	UNP P0DTC2
C	1245	ARG	-	expression tag	UNP P0DTC2
C	1246	SER	-	expression tag	UNP P0DTC2
C	1247	LEU	-	expression tag	UNP P0DTC2
C	1248	GLU	-	expression tag	UNP P0DTC2
C	1249	VAL	-	expression tag	UNP P0DTC2
C	1250	LEU	-	expression tag	UNP P0DTC2
C	1251	PHE	-	expression tag	UNP P0DTC2
C	1252	GLN	-	expression tag	UNP P0DTC2
C	1253	GLY	-	expression tag	UNP P0DTC2
C	1254	PRO	-	expression tag	UNP P0DTC2
C	1255	GLY	-	expression tag	UNP P0DTC2
C	1256	TRP	-	expression tag	UNP P0DTC2
C	1257	SER	-	expression tag	UNP P0DTC2
C	1258	HIS	-	expression tag	UNP P0DTC2
C	1259	PRO	-	expression tag	UNP P0DTC2
C	1260	GLN	-	expression tag	UNP P0DTC2
C	1261	PHE	-	expression tag	UNP P0DTC2
C	1262	GLU	-	expression tag	UNP P0DTC2
C	1263	LYS	-	expression tag	UNP P0DTC2
C	1264	GLY	-	expression tag	UNP P0DTC2
C	1265	GLY	-	expression tag	UNP P0DTC2
C	1266	GLY	-	expression tag	UNP P0DTC2
C	1267	SER	-	expression tag	UNP P0DTC2
C	1268	GLY	-	expression tag	UNP P0DTC2
C	1269	GLY	-	expression tag	UNP P0DTC2
C	1270	GLY	-	expression tag	UNP P0DTC2
C	1271	SER	-	expression tag	UNP P0DTC2
C	1272	GLY	-	expression tag	UNP P0DTC2
C	1273	GLY	-	expression tag	UNP P0DTC2
C	1274	SER	-	expression tag	UNP P0DTC2
C	1275	SER	-	expression tag	UNP P0DTC2
C	1276	ALA	-	expression tag	UNP P0DTC2
C	1277	TRP	-	expression tag	UNP P0DTC2
C	1278	SER	-	expression tag	UNP P0DTC2
C	1279	HIS	-	expression tag	UNP P0DTC2
C	1280	PRO	-	expression tag	UNP P0DTC2

Continued on next page...

For Manuscript Review

Continued from previous page...

Chain	Residue	Modelled	Actual	Comment	Reference
C	1281	GLN	-	expression tag	UNP P0DTC2
C	1282	PHE	-	expression tag	UNP P0DTC2
C	1283	GLU	-	expression tag	UNP P0DTC2
C	1284	LYS	-	expression tag	UNP P0DTC2
C	1285	HIS	-	expression tag	UNP P0DTC2
C	1286	HIS	-	expression tag	UNP P0DTC2
C	1287	HIS	-	expression tag	UNP P0DTC2
C	1288	HIS	-	expression tag	UNP P0DTC2
C	1289	HIS	-	expression tag	UNP P0DTC2
C	1290	HIS	-	expression tag	UNP P0DTC2
C	1291	HIS	-	expression tag	UNP P0DTC2
C	1292	HIS	-	expression tag	UNP P0DTC2

- Molecule 2 is a protein called CR9 heavy chain.

Mol	Chain	Residues	Atoms					AltConf	Trace
2	H	115	Total 863	C 540	N 152	O 165	S 6	0	0
2	G	115	Total 863	C 540	N 152	O 165	S 6	0	0
2	I	115	Total 863	C 540	N 152	O 165	S 6	0	0

- Molecule 3 is a protein called CR9 light chain.

Mol	Chain	Residues	Atoms					AltConf	Trace
3	L	107	Total 816	C 510	N 139	O 165	S 2	0	0
3	J	107	Total 816	C 510	N 139	O 165	S 2	0	0
3	K	107	Total 816	C 510	N 139	O 165	S 2	0	0

For Manuscript Review

3 Residue-property plots (i)

These plots are drawn for all protein, RNA, DNA and oligosaccharide chains in the entry. The first graphic for a chain summarises the proportions of the various outlier classes displayed in the second graphic. The second graphic shows the sequence view annotated by issues in geometry and atom inclusion in map density. Residues are color-coded according to the number of geometric quality criteria for which they contain at least one outlier: green = 0, yellow = 1, orange = 2 and red = 3 or more. A red diamond above a residue indicates a poor fit to the EM map for this residue (all-atom inclusion < 40%). Stretches of 2 or more consecutive residues without any outlier are shown as a green connector. Residues present in the sample, but not in the model, are shown in grey.

- Molecule 1: Spike glycoprotein

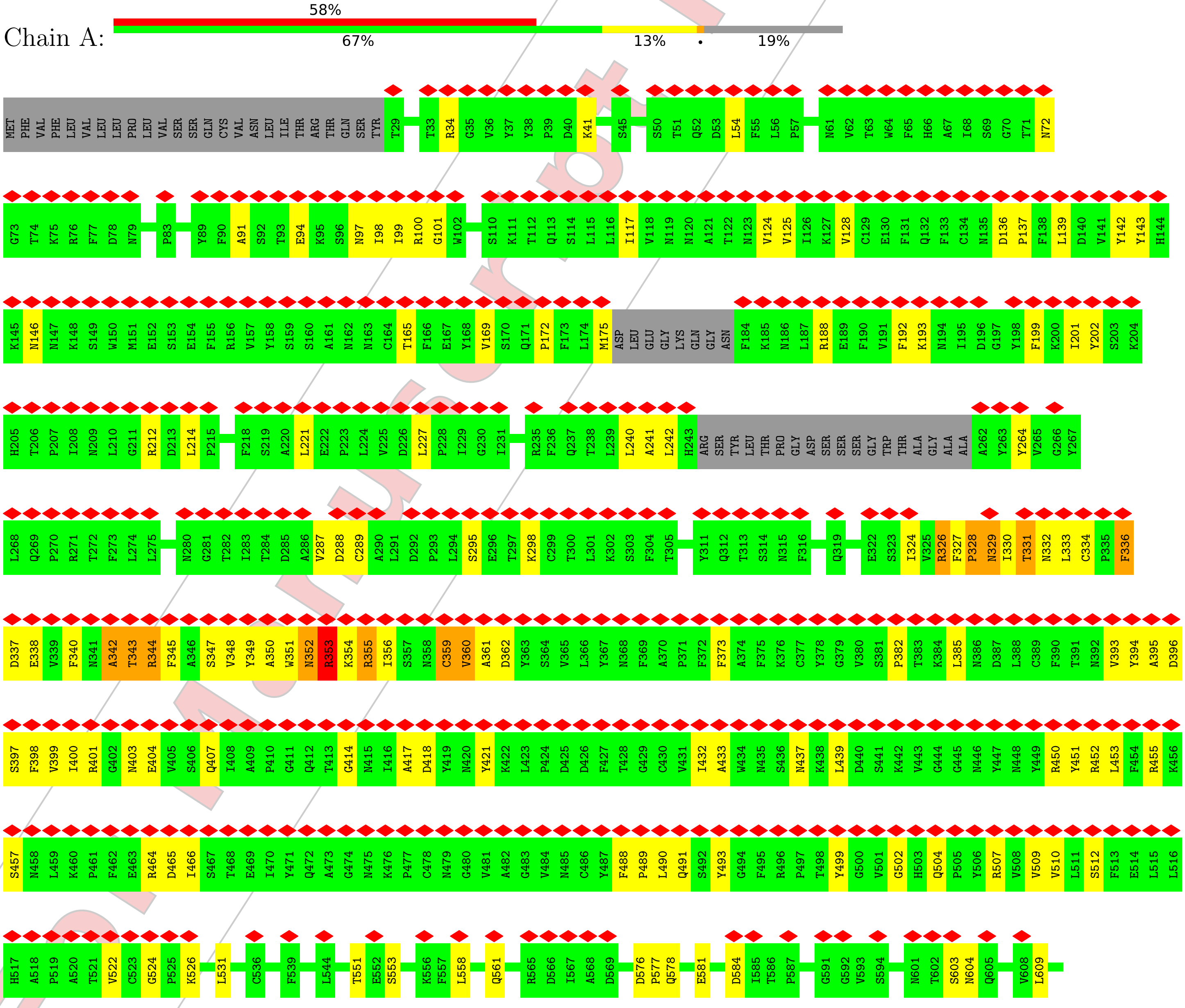

For Manuscript Review

C615 V618 P619 V620 A621 ILE HIS ALA ASP GLN LEU THR PRO THR TRP ARG VAL TYR SER THR G637 S638 N639 V640 F641 Q642 T643 R644 A645 L648 A651 N655 S656 S657 Y658 E659 C660 D661 G667 S671 Y672 Q673 T674 Q675 THR LYS SER HIS ARG ALA ALA ALA SER VAL

ALA S687 Y693 T694 M695 S696 L697 G698 A699 E700 N701 S702 V703 A704 Y705 S706 N707 N708 S709 I710 A711 I712 P713 T714 N715 F716 T717 V720 T721 T722 E723 I724 V727 S728 M729 S733 V734 D735 C736 T737 M738 C741 G742 D743 S744 T745 E746 C747 S748 N749 L750 L751 L752 Q753 Y754

G755 S756 F757 C758 T759 Q760 R763 A764 G767 I768 E771 Q772 D773 K774 N775 E778 V779 F780 A781 Q782 Q785 I786 Y787 P791 Y794 F795 G796 G797 F798 N799 F800 S801 Q802 I803 L804 P805 D806 P807 S808 K809 S814 P815 K823 L826 ALA ASP ALA GLY PHE ILE

LYS GLN TYR GLY ASP CYS LEU GLY ASP ILE ALA ALA ARG ASP L847 I848 C849 A850 Q851 K852 F853 L856 P861 L862 L863 T864 D865 E866 M867 I868 A869 Q870 S873 A874 A877 I880 T881 W884 G889 L892 P895 F896 P897 M900 R903 F904 N905 G906 I907

G908 V909 T910 V913 N917 L920 I921 F925 N926 S927 A928 I929 I932 S935 L936 S937 S938 L943 G944 Q947 N953 L957 N958 T959 L960 V961 K962 Q963 L964 S965 S966 K967 F968 G969 A970 I971 S972 S973 V974 L975 N976 D977 I978 L979 S980 R981 L982 D983 P984 P985

E986 A987 E988 V989 Q990 I991 D992 R993 L994 I995 T996 G997 R998 L999 Q1000 S1001 L1002 Q1003 V1006 T1007 Q1008 Q1009 L1010 I1011 E1015 A1018 S1019 A1020 N1021 L1022 A1023 S1028 S1035 K1036 R1037 V1038 D1039 F1040 C1041 G1042 K1043 G1044 Y1045 H1046 L1047 M1048 S1049 F1050 P1051 Q1052 S1053 A1054 P1055 H1056 G1057

V1058 V1059 F1060 L1061 H1062 V1063 T1064 Y1065 V1066 P1067 A1068 K1071 N1072 F1073 T1074 T1075 A1076 P1077 A1078 I1079 C1080 H1081 D1082 G1083 K1084 A1085 H1086 F1087 P1088 R1089 E1090 G1091 V1092 F1093 V1094 S1095 N1096 G1097 W1100 F1101 V1102 T1103 Q1104 R1105 N1106 F1107 Y1108 E1109 P1110 Q1111 I1112 I1113 T1114 T1115 D1116 N1117 T1118 F1119

V1120 S1121 G1122 N1123 C1124 D1125 V1126 V1127 I1128 G1129 I1130 V1131 N1132 N1133 T1134 V1135 Y1136 D1137 P1138 L1139 Q1140 P1141 E1142 L1143 D1144 S1145 F1146 K1147 E1148 GLU LEU ASP LYS TYR PHE LYS ASN HIS THR SER PRO ASP VAL ASP LEU GLY ASP ILE SER GLY ILE ASN ALA SER VAL VAL ASN ILE GLN LYS

GLU ILE ASP ARG LEU ASN GLU VAL ALA LYS ASN LEU ASN GLU SER LEU ILE ASP LEU GLN GLU LEU GLY LYS TYR GLU GLN TYR ILE LYS TRP PRO GLY GLY SER GLY GLY SER TYR ILE PRO GLU ALA PRO ARG ASP GLY GLN ALA TYR VAL ARG LYS ASP GLY GLU TRP VAL LEU LEU

SER THR PHE LEU GLY ARG SER LEU GLU VAL LEU PHE GLN GLY PRO GLY TRP SER HIS PRO GLN PHE GLU LYS GLY GLY GLY SER GLY GLY GLY SER GLY GLY SER ALA TRP SER HIS PRO GLN PHE GLU LYS HIS HIS HIS HIS HIS HIS HIS HIS

- Molecule 1: Spike glycoprotein

Chain B: 69% | 70% | 11% | 18%

MET PHE VAL PHE LEU VAL LEU LEU PRO LEU VAL SER SER GLN CYS VAL ASN LEU ILE THR ARG THR GLN SER TYR T29 N30 S31 F32 T33 R34 G35 V36 Y37 Y38 P39 D40 K41 V42 F43 R44 S45 S46 V47 L48 H49 S50 T51 Q52 D53 L54 F55 L56 P57 F58 F59 S60 N61 V62 T63

W64 F65 H66 A67 I68 S69 G70 T71 N72 G73 T74 K75 R76 F77 D78 N79 P80 V81 L82 P83 F84 N85 D86 G87 V88 Y89 F90 A91 S92 T93 E94 K95 S96 N97 I98 I99 R100 G101 W102 I103 F104 G105 T106 T107 L108 D109 S110 K111 T112 Q113 S114 L115 L116 I117 V118 N119 N120 A121 T122 N123

V124 V125 I126 K127 V128 C129 E130 F131 Q132 F133 C134 N135 D136 P137 F138 L139 D140 V141 Y142 Y143 H144 K145 N146 N147 K148 S149 W150 M151 E152 S153 E154 F155 R156 V157 Y158 S159 S160 A161 N162 N163 C164 T165 F166 E167 Y168 V169 S170 Q171 P172 F173 L174 M175 D176 L177 E178 G179 K180 Q181 G182 N183

For Manuscript Review

F184 K185 N186 L187 R188 E189 F190 V191 F192 K193 N194 I195 D196 G197 Y198 F199 K200 I201 Y202 S203 K204 H205 T206 P207 I208 N209 L210 G211 R212 D213 L214 P215 Q216 G217 F218 S219 A220 L221 E222 P223 L224 V225 D226 L227 P228 I229 G230 I231 N232 I233 T234 R235 F236 Q237 T238 L239 L240 A241 L242 H243

ARG SER TYR LEU THR PRO GLY ASP SER SER GLY TRP THR ALA GLY ALA ALA A262 Y263 Y264 V265 G266 Y267 L268 Q269 P270 R271 T272 F273 L274 L275 K276 Y277 N278 E279 N280 G281 T282 I283 T284 D285 A286 V287 D288 C289 A290 L291 D292 P293 L294 S295 E296 T297 K298 C299 T300 L301 K302 S303

F304 T305 V306 E307 K308 G309 I310 Y311 Q312 T313 S314 N315 F316 R317 V318 Q319 P320 T321 E322 S323 I324 V325 R326 F327 P328 N329 I330 T331 N332 L333 C334 P335 F336 D337 E338 V339 F340 N341 A342 T343 R344 F345 A346 S347 V348 Y349 A350 W351 N352 R353 K354 R355 I356 S357 N358 C359 V360 A361 D362 Y363

S364 V365 L366 Y367 N368 F369 A370 P371 F372 F373 A374 C377 Y378 S381 P382 T383 K384 L385 T391 N392 V393 Y394 A395 D396 S397 F398 V399 I400 R401 E404 V405 S406 Q407 I408 A409 Q412 N415 I416 A417 D418 Y419 N420 Y421 K422 L423 P424 D425 D426 F427 T428 G429 C430 A433

W434 N435 S436 N437 K438 L439 D440 S441 K442 V443 G444 G445 N446 Y447 N448 Y449 R450 Y451 R452 L453 F454 R455 K456 S457 N458 L459 K460 P461 F462 E463 R464 D465 I466 S467 T468 E469 I470 Y471 Q472 A473 G474 N475 K476 P477 C478 N479 G480 V481 A482 G483 V484 N485 C486 Y487 F488 P489 L490 Q491 S492 Y493

G494 F495 R496 P497 T498 Y499 G500 V501 G502 H503 Q504 P505 Y506 R507 V508 S512 F513 E514 L515 L516 H517 A518 P519 A520 T521 V522 C523 G524 P525 K526 K527 S528 T529 N530 L531 V532 K533 N534 K535 C536 V537 N538 F539 N540 F541 N542 T547 G548 V549 L550 T551 E552 S553 N554 K555 K556 F557 L558

P559 F560 Q561 Q562 F563 G564 R565 D566 T570 V574 R575 D576 P577 Q578 T579 L580 E581 I582 L583 T586 P587 C588 S589 F590 G591 G592 V593 S594 V595 I596 T597 P598 G599 T600 N601 T602 S603 N604 Q605 V606 A607 V608 L609 Y610 Q611 G612 V613 N614 C615 T616 E617 V618 P619 V620 ALA ILE HIS

ALA ASP GLN LEU THR PRO THR TRP ARG VAL TYR SER THR G637 S638 N639 V640 F641 Q642 T643 R644 A645 G646 C647 L648 I649 G650 A651 E652 Y653 V654 N655 S656 S657 Y658 E659 C660 D661 I662 P663 I664 G665 A666 G667 I668 C669 A670 S671 Y672 Q673 T674 Q675 THR LYS SER HIS ARG ALA ALA ALA

SER VAL ALA S687 Q688 S689 I690 I691 A692 Y693 T694 M695 S696 L697 G698 A699 E700 N701 S702 V703 A704 Y705 S706 N707 N708 S709 I710 A711 I712 P713 T714 N715 F716 T717 I718 S719 T722 E723 I724 D735 C736 T737 M738 Y739 I740 C741 G742 D743 C747 S748 N749 L750 L751 L752 Q753 Y754 G755

S756 F757 C758 T759 Q760 L761 K762 R763 A764 L765 T766 G767 I768 A769 V770 E771 Q772 V783 K784 Q785 I786 Y787 K788 T789 P790 P791 I792 K793 Y794 F795 G796 G797 F798 N799 F800 S801 Q802 I803 L804 P805 D806 P807 S808 K809 P810 S811 K812 R813 S814 P815 I816 E817 D818 L819 L820 F821 N822 K823 V824

T825 L826 ALA ASP ALA GLY PHE ILE LYS GLN TYR GLY ASP CYS LEU GLY ASP ILE ALA ALA ARG ASP L847 I848 C849 A850 Q851 K852 F853 N854 G855 P860 T864 D865 E866 M867 I868 A869 Q870 Y871 T872 S873 A874 L875 L876 A877 G878 T879 I880 T881 S882 G883 W884 T885 F886 G887 A888

A891 L892 Q893 I894 P895 F896 P897 M898 Q899 M900 A901 Y902 R903 F904 N905 G906 I907 G908 V909 T910 Q911 N912 V913 L914 Y915 E916 N917 Q918 K919 L920 I921 A922 N923 Q924 F925 N926 S927 G930 K931 I932 Q933 D934 S935 L936 S937 S938 T939 P940 S941 A942 Q947 N951 H952 N953 A954 Q955 A956

L957 N958 T959 L960 V961 K962 Q963 L964 S965 S966 K967 F968 G969 A970 I971 S972 S973 V974 D977 I978 L979 S980 R981 L982 D983 P984 P985 E986 A987 E988 V989 Q990 I991 D992 R993 L994 I995 T996 G997 R998 L999 Q1000 S1001 L1002 Q1003 T1004 Y1005 V1006 T1007 Q1008 Q1009 L1010 I1011 R1012 A1013 A1014 E1015 I1016 R1017

For Manuscript Review

L1022 K1026 M1027 S1028 V1031 L1032 G1033 Y1045 H1046 L1047 S1053 A1054 P1055 H1056 G1057 T1064 Y1065 V1066 P1067 A1068 Q1069 E1070 K1071 N1072 F1073 T1074 T1075 A1076 P1077 A1078 I1079 C1080 H1081 D1082 G1083 K1084 A1085 H1086 F1087 P1088 G1091 V1092 F1093 V1094 S1095 N1096 G1097 T1098 H1099 W1100 F1101 V1102 T1103

Q1104 R1105 N1106 F1107 Y1108 E1109 P1110 Q1111 I1112 I1113 T1114 T1115 D1116 N1117 T1118 F1119 V1120 N1123 C1124 D1125 V1126 V1127 I1128 G1129 I1130 V1131 N1132 N1133 T1134 V1135 Y1136 D1137 P1138 L1139 Q1140 P1141 E1142 L1143 D1144 S1145 F1146 K1147 E1148 GLU LEU ASP LYS TYR PHE LYS ASN HIS THR SER PRO ASP VAL ASP LEU

GLY ASP ILE SER GLY ILE ASN ALA SER VAL VAL ASN ILE GLN LYS GLU ILE ASP ARG LEU ASN GLU VAL ALA LYS ASN LEU ASN GLU SER LEU ILE ASP LEU GLN GLU LEU GLY LYS TYR GLU GLN TYR ILE LYS TRP PRO GLY GLY SER GLY GLY SER TYR ILE PRO GLU ALA PRO ARG

ASP GLY GLN ALA TYR VAL ARG LYS ASP GLY GLU TRP VAL LEU LEU SER THR PHE LEU GLY ARG SER LEU GLU VAL LEU PHE GLN GLY PRO GLY TRP SER HIS PRO GLN PHE GLU LYS GLY GLY GLY SER GLY GLY GLY SER GLY GLY SER SER ALA TRP SER HIS PRO GLN PHE GLU LYS

HIS HIS HIS HIS HIS HIS HIS HIS

- Molecule 1: Spike glycoprotein

Chain C: 58% | 71% | 11% | 19%

MET PHE VAL PHE LEU VAL LEU LEU PRO LEU VAL SER SER GLN CYS VAL ASN LEU ILE THR ARG THR GLN SER TYR T29 N30 S31 F32 G35 V36 Y37 Y38 P39 D40 K41 V42 F43 R44 S45 S46 V47 L48 H49 S50 T51 Q52 D53 L54 F55 L56 N61 W64 A67 I68 S69

G70 T71 N72 G73 T74 K75 R76 F77 D78 V81 N85 D86 F90 A91 S92 T93 E94 K95 R100 G101 W102 I103 F104 G105 T106 T107 L108 D109 S110 K111 T112 Q113 S114 L115 L116 I117 V118 N119 N120 A121 V124 V125 I126 K127 V128 C129 E130 F131 Q132 F133 C134 N135 D136 P137 F138

L139 D140 V141 Y142 Y143 H144 K145 N146 N147 K148 S149 W150 M151 E152 S153 E154 F155 R156 V157 Y158 S159 S160 A161 N162 N163 C164 T165 F166 E167 Y168 V169 S170 Q171 P172 F173 L174 M175 D176 L177 E178 G179 K180 Q181 F184 K185 N186 L187 R188 E189 K193 N194 I195 D196 G197 Y198 F199 K200 I201

Y202 S203 K204 H205 T206 P207 R212 D213 L214 P215 S219 A220 P223 L224 V225 D226 L227 P228 I229 G230 I231 N232 I233 T234 R235 L239 L240 A241 L242 H243 R244 SER TYR LEU THR PRO GLY ASP SER SER SER GLY TRP THR ALA GLY ALA A261 Y264 P270 R271 T272 F273 L274

L275 K276 Y277 N278 E279 N280 G281 T282 I283 T284 V287 D288 C289 A290 L291 D292 P293 L294 S295 E296 T297 K298 C299 T300 L301 K302 S303 F304 T305 G309 I310 Y311 Q312 T313 S314 N315 F316 R317 V318 Q319 P320 T321 E322 S323 I324 V325 R326 F327 P328 N329 I330 T331 N332 L333 C334 P335 F336 D337

E338 V339 F340 N341 A342 T343 R344 F345 A346 S347 V348 Y349 A350 W351 N352 R353 K354 R355 I356 S357 N358 C359 V360 A361 D362 Y363 S364 V365 L366 Y367 N368 F369 A370 P371 F372 F373 A374 F375 K376 C377 Y378 G379 V380 S381 P382 T383 K384 L385 N386 D387 L388 C389 F390 T391 N392 V393 Y394 A395 D396 S397

F398 V399 I400 R401 G402 N403 E404 V405 S406 Q407 I408 A409 P410 G411 Q412 T413 G414 N415 I416 A417 D418 Y419 N420 Y421 K422 L423 P424 D425 D426 F427 T428 G429 C430 V431 I432 A433 W434 N435 S436 N437 K438 L439 D440 V443 G444 G445 N446 Y447 N448 Y449 R450 Y451 R452 L453 F454 R455 K456 S457 N458

L459 K460 P461 F462 E463 R464 D465 I466 S467 T468 E469 I470 Y471 Q472 A473 G474 N475 K476 P477 C478 N479 G480 V481 A482 G483 V484 N485 C486 Y487 F488 P489 L490 Q491 S492 Y493 G494 F495 R496 Y499 Q504 P505 Y506 R507 V508 V509 V510 L511 S512 F513 E514 L515 L516 H517 A518 P519 A520 T521 V522

For Manuscript Review

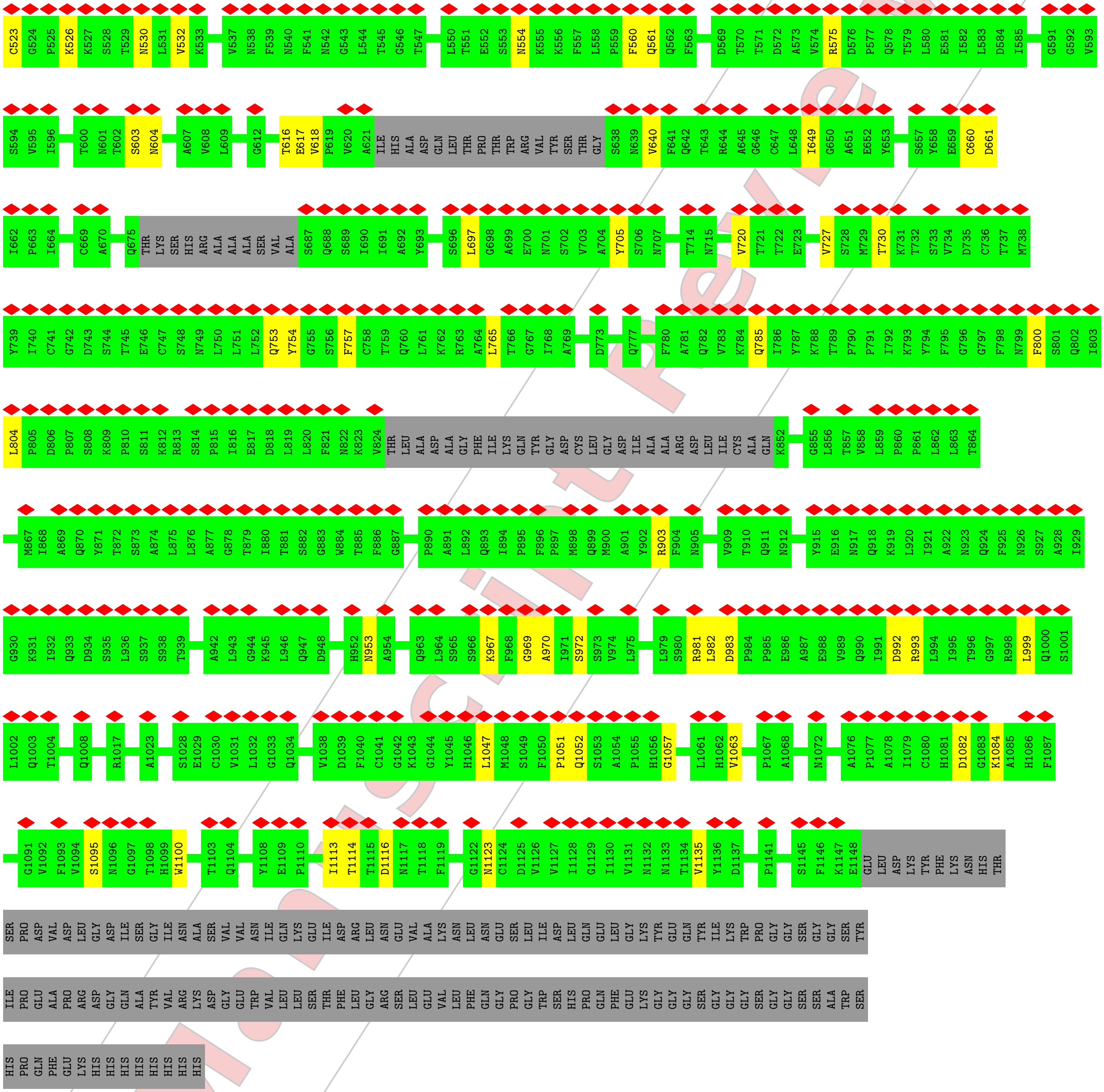

• Molecule 2: CR9 heavy chain

Chain H:

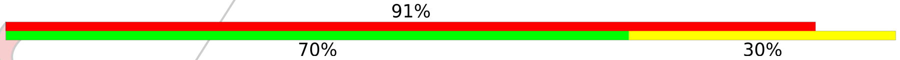

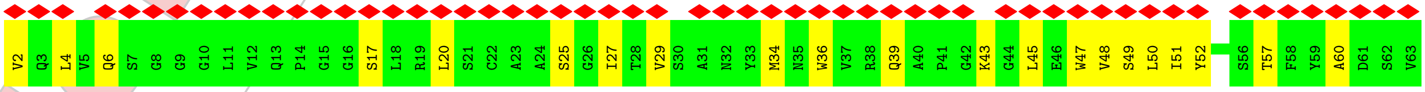

For Manuscript Review

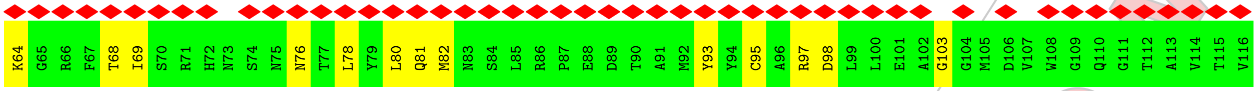

- Molecule 2: CR9 heavy chain

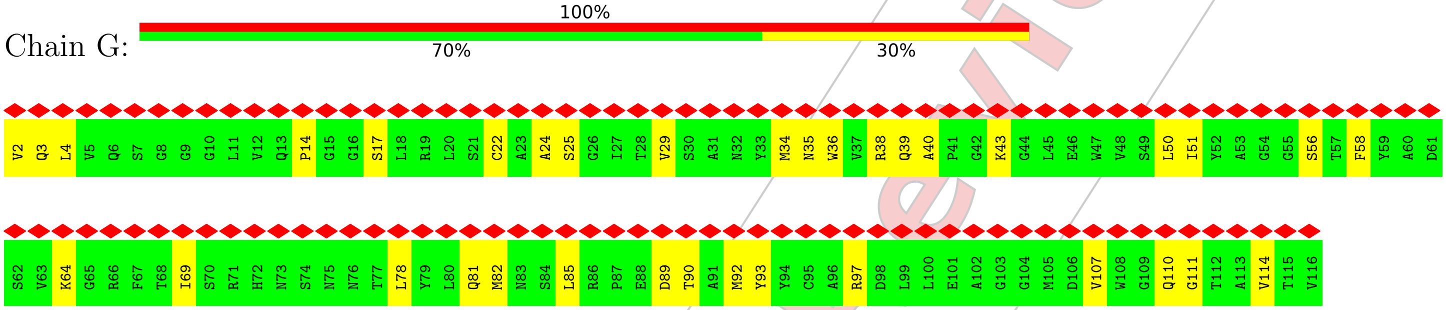

- Molecule 2: CR9 heavy chain

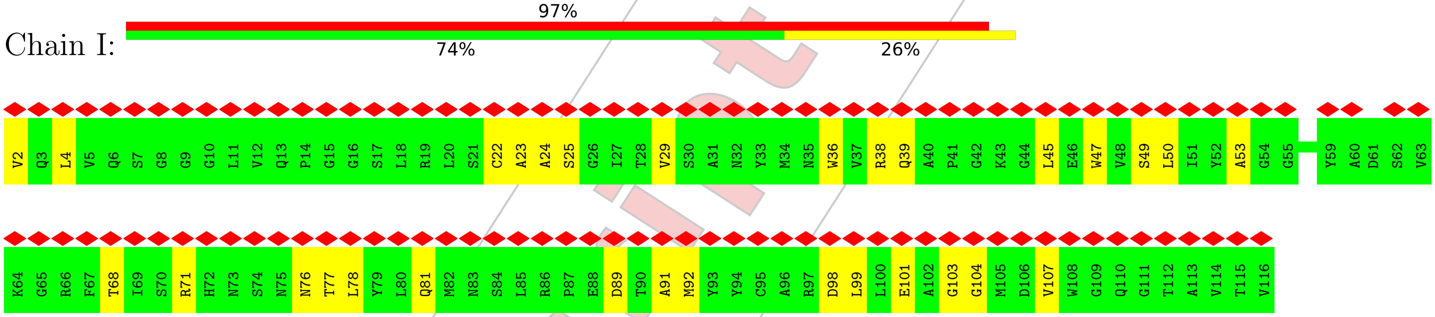

- Molecule 3: CR9 light chain

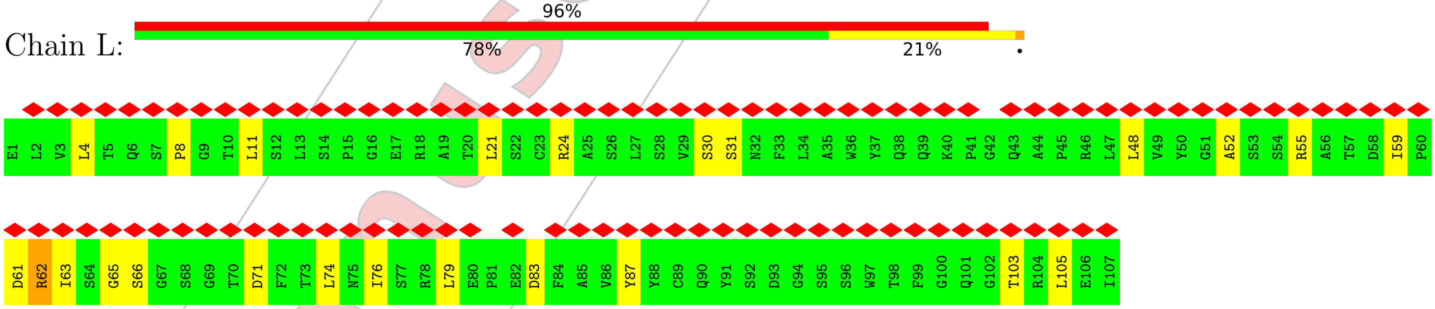

- Molecule 3: CR9 light chain

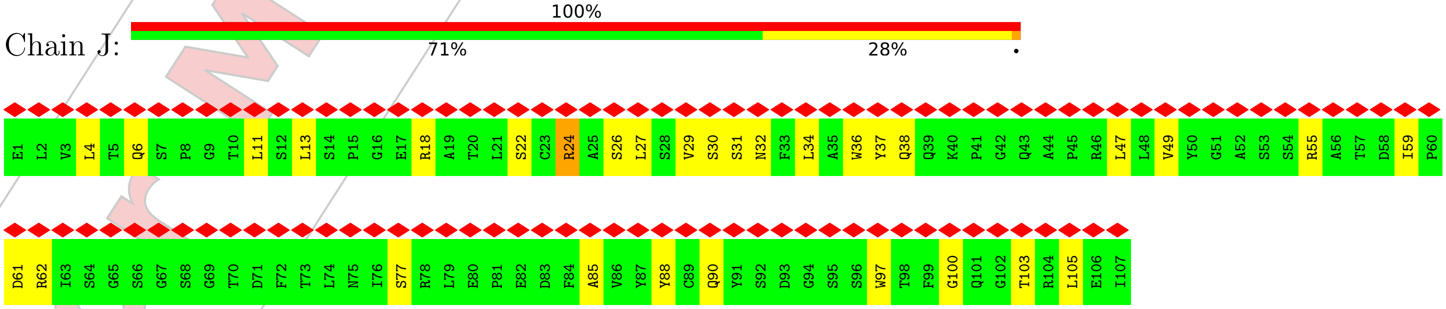

- Molecule 3: CR9 light chain

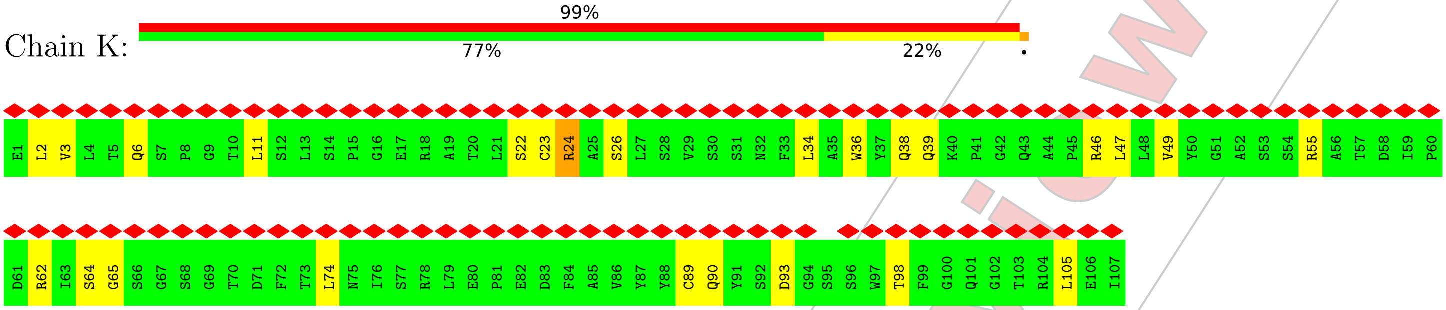

For Manuscript Review

4 Experimental information ⓘ

Property	Value	Source
EM reconstruction method	SINGLE PARTICLE	Depositor
Imposed symmetry	POINT, Not provided	
Number of particles used	282590	Depositor
Resolution determination method	DIFFRACTION PATTERN/LAYERLINES	Depositor
CTF correction method	PHASE FLIPPING AND AMPLITUDE CORRECTION	Depositor
Microscope	FEI TITAN KRIOS	Depositor
Voltage (kV)	300	Depositor
Electron dose ($e^-/\text{Å}^2$)	52	Depositor
Minimum defocus (nm)	1200	Depositor
Maximum defocus (nm)	1800	Depositor
Magnification	Not provided	
Image detector	FEI FALCON IV (4k x 4k)	Depositor
Maximum map value	2.716	Depositor
Minimum map value	-1.480	Depositor
Average map value	-0.001	Depositor
Map value standard deviation	0.078	Depositor
Recommended contour level	0.19	Depositor
Map size (Å)	342.40002, 342.40002, 342.40002	wwPDB
Map dimensions	320, 320, 320	wwPDB
Map angles (°)	90.0, 90.0, 90.0	wwPDB
Pixel spacing (Å)	1.07, 1.07, 1.07	Depositor

For Manuscript Review

5 Model quality (i)

5.1 Standard geometry (i)

The Z score for a bond length (or angle) is the number of standard deviations the observed value is removed from the expected value. A bond length (or angle) with $|Z| > 5$ is considered an outlier worth inspection. RMSZ is the root-mean-square of all Z scores of the bond lengths (or angles).

Mol	Chain	Bond lengths		Bond angles	
		RMSZ	#$|Z|$ >5	RMSZ	#$|Z|$ >5
1	A	0.30	0/8397	0.55	1/11428 (0.0%)
1	B	0.28	0/8452	0.54	0/11502
1	C	0.27	0/8412	0.54	0/11448
2	G	0.26	0/880	0.62	0/1194
2	H	0.27	0/880	0.63	0/1194
2	I	0.26	0/880	0.59	0/1194
3	J	0.26	0/834	0.63	0/1134
3	K	0.26	0/834	0.63	0/1134
3	L	0.27	0/834	0.62	0/1134
All	All	0.28	0/30403	0.56	1/41362 (0.0%)

Chiral center outliers are detected by calculating the chiral volume of a chiral center and verifying if the center is modelled as a planar moiety or with the opposite hand.A planarity outlier is detected by checking planarity of atoms in a peptide group, atoms in a mainchain group or atoms of a sidechain that are expected to be planar.

Mol	Chain	#Chirality outliers	#Planarity outliers
1	A	0	4
1	B	0	1
1	C	0	1
All	All	0	6

There are no bond length outliers.

All (1) bond angle outliers are listed below:

Mol	Chain	Res	Type	Atoms	Z	Observed(^{o})	Ideal(^{o})
1	A	139	LEU	CA-CB-CG	6.75	130.83	115.30

There are no chirality outliers.

All (6) planarity outliers are listed below:

For Manuscript Review

Mol	Chain	Res	Type	Group
1	A	326	ARG	Sidechain
1	A	329	ASN	Peptide
1	A	344	ARG	Sidechain
1	A	353	ARG	Sidechain
1	B	326	ARG	Sidechain
1	C	329	ASN	Peptide

5.2 Too-close contacts ⓘ

In the following table, the Non-H and H(model) columns list the number of non-hydrogen atoms and hydrogen atoms in the chain respectively. The H(added) column lists the number of hydrogen atoms added and optimized by MolProbity. The Clashes column lists the number of clashes within the asymmetric unit, whereas Symm-Clashes lists symmetry-related clashes.

Mol	Chain	Non-H	H(model)	H(added)	Clashes	Symm-Clashes
1	A	8201	0	8008	142	0
1	B	8255	0	8060	102	0
1	C	8215	0	8009	88	0
2	G	863	0	838	23	0
2	H	863	0	838	21	0
2	I	863	0	838	19	0
3	J	816	0	785	18	0
3	K	816	0	785	16	0
3	L	816	0	785	13	0
All	All	29708	0	28946	418	0

The all-atom clashscore is defined as the number of clashes found per 1000 atoms (including hydrogen atoms). The all-atom clashscore for this structure is 7.

All (418) close contacts within the same asymmetric unit are listed below, sorted by their clash magnitude.

Atom-1	Atom-2	Interatomic distance (Å)	Clash overlap (Å)
1:A:348:VAL:HG21	1:A:493:TYR:HE2	1.27	1.00
1:A:329:ASN:HB2	1:A:578:GLN:HA	1.41	0.97
1:A:348:VAL:HB	1:A:451:TYR:HB3	1.45	0.96
1:B:331:THR:HG21	1:B:525:PRO:HG3	1.54	0.89
1:A:348:VAL:HB	1:A:451:TYR:CB	2.05	0.86
1:A:360:VAL:HA	1:A:522:VAL:HG12	1.56	0.83
3:L:4:LEU:HA	3:L:24:ARG:O	1.77	0.83
1:A:352:ASN:H	1:A:398:PHE:HB3	1.44	0.81
1:A:348:VAL:HG21	1:A:493:TYR:CE2	2.14	0.80

Continued on next page...

For Manuscript Review

Continued from previous page...

Atom-1	Atom-2	Interatomic distance (Å)	Clash overlap (Å)
2:G:17:SER:HA	2:G:82:MET:O	1.81	0.79
2:H:17:SER:HA	2:H:82:MET:O	1.84	0.78
1:A:576:ASP:HB2	1:A:581:GLU:H	1.55	0.72
1:A:348:VAL:CB	1:A:451:TYR:HB3	2.20	0.71
1:A:394:TYR:HB2	1:A:512:SER:HB3	1.73	0.71
1:A:753:GLN:HE22	1:C:969:GLY:H	1.37	0.71
1:B:326:ARG:O	1:B:577:PRO:HG2	1.91	0.71
1:A:360:VAL:CA	1:A:522:VAL:HG12	2.21	0.70
1:A:344:ARG:HH22	1:A:439:LEU:HG	1.57	0.70
1:A:199:PHE:HB3	1:A:227:LEU:HB2	1.76	0.67
1:C:450:ARG:HE	1:C:490:LEU:HB3	1.56	0.67
1:A:334:CYS:SG	1:A:360:VAL:N	2.67	0.67
1:B:326:ARG:HE	1:B:578:GLN:HE21	1.43	0.67
1:C:135:ASN:HB2	1:C:157:VAL:HA	1.76	0.66
3:J:59:ILE:HG22	3:J:61:ASP:H	1.60	0.65
1:A:329:ASN:CB	1:A:578:GLN:HA	2.24	0.64
3:L:21:LEU:HD11	3:L:103:THR:HG21	1.80	0.64
1:A:326:ARG:HG3	1:A:531:LEU:HD12	1.79	0.63
1:B:331:THR:CG2	1:B:525:PRO:HG3	2.28	0.63
2:I:36:TRP:HB3	2:I:49:SER:HB2	1.81	0.63
2:I:39:GLN:HB3	2:I:92:MET:HB2	1.80	0.63
1:A:354:LYS:N	1:A:395:ALA:O	2.32	0.63
1:B:441:SER:HB2	1:B:497:PRO:HB3	1.79	0.63
1:B:326:ARG:HD3	1:B:528:SER:O	2.00	0.62
1:A:334:CYS:HA	1:A:359:CYS:HB2	1.82	0.62
1:B:326:ARG:HE	1:B:578:GLN:NE2	1.97	0.62
1:B:331:THR:HB	1:B:360:VAL:HG21	1.82	0.62
1:A:352:ASN:HB2	1:A:397:SER:O	2.00	0.62
3:L:8:PRO:O	3:L:103:THR:OG1	2.19	0.61
2:G:38:ARG:NH2	2:G:89:ASP:O	2.34	0.61
3:J:37:TYR:HB3	3:J:47:LEU:HD13	1.82	0.61
1:A:354:LYS:HB2	1:A:395:ALA:HB3	1.82	0.60
1:A:356:ILE:HB	1:A:393:VAL:HB	1.82	0.60
1:A:351:TRP:CD1	1:A:421:TYR:HB2	2.36	0.60
1:A:404:GLU:HG3	1:A:407:GLN:HE21	1.67	0.60
1:B:576:ASP:HB3	1:B:578:GLN:O	2.01	0.60
1:A:137:PRO:HG3	1:A:241:ALA:HA	1.83	0.60
1:A:72:ASN:ND2	1:A:142:TYR:O	2.34	0.60
1:B:450:ARG:NH2	1:B:489:PRO:O	2.35	0.60
2:H:68:THR:HB	2:H:81:GLN:HB3	1.83	0.60
1:B:113:GLN:NE2	1:B:128:VAL:O	2.35	0.60

Continued on next page...

For Manuscript Review

Continued from previous page...

Atom-1	Atom-2	Interatomic distance (Å)	Clash overlap (Å)
1:C:903:ARG:NH1	1:C:1047:LEU:O	2.35	0.60
1:A:1089:ARG:NH1	1:A:1116:ASP:O	2.35	0.60
1:A:551:THR:HG23	1:A:584:ASP:HB3	1.83	0.60
1:B:135:ASN:ND2	1:B:156:ARG:O	2.34	0.59
3:L:62:ARG:NH2	3:L:83:ASP:OD2	2.35	0.59
3:J:36:TRP:HB2	3:J:49:VAL:HG12	1.84	0.59
1:C:970:ALA:HB2	1:C:993:ARG:HD2	1.85	0.59
1:A:326:ARG:O	1:A:577:PRO:HG2	2.03	0.59
2:H:27:ILE:O	2:H:76:ASN:ND2	2.35	0.59
2:H:36:TRP:HB3	2:H:49:SER:HB2	1.85	0.59
1:B:491:GLN:NE2	2:I:101:GLU:OE2	2.36	0.58
1:B:579:THR:O	1:B:581:GLU:HG3	2.03	0.58
2:G:92:MET:HG3	2:G:111:GLY:HA2	1.86	0.58
1:B:578:GLN:C	1:B:580:LEU:H	2.07	0.58
1:A:450:ARG:NH2	1:A:489:PRO:O	2.36	0.58
1:C:324:ILE:HD11	1:C:532:VAL:H	1.69	0.58
1:A:97:ASN:O	1:A:100:ARG:NH1	2.37	0.57
2:H:39:GLN:NE2	2:H:43:LYS:O	2.37	0.57
1:B:800:PHE:HB3	1:B:804:LEU:HD23	1.87	0.57
1:B:417:ALA:HB1	1:B:422:LYS:HD3	1.86	0.57
1:B:124:VAL:HG13	1:B:172:PRO:HA	1.86	0.56
2:I:47:TRP:HE1	2:I:50:LEU:HG	1.71	0.56
1:C:101:GLY:HA3	1:C:239:LEU:HB3	1.88	0.56
1:C:104:PHE:HB2	1:C:115:LEU:HB2	1.87	0.56
3:L:48:LEU:HD11	3:L:87:TYR:HD2	1.71	0.56
1:C:327:PHE:HB3	1:C:328:PRO:HD2	1.86	0.56
1:B:199:PHE:HB3	1:B:227:LEU:HB2	1.88	0.56
3:L:59:ILE:HG22	3:L:61:ASP:H	1.71	0.56
1:C:355:ARG:HH12	1:C:392:ASN:HB2	1.71	0.56
1:A:499:TYR:HE1	3:J:29:VAL:HA	1.70	0.55
2:G:90:THR:HG23	2:G:114:VAL:H	1.70	0.55
1:B:412:GLN:O	1:B:422:LYS:NZ	2.38	0.55
3:J:18:ARG:HG2	3:J:77:SER:HA	1.89	0.55
1:B:847:LEU:HG	1:B:849:CYS:H	1.71	0.55
1:C:660:CYS:SG	1:C:661:ASP:N	2.80	0.55
2:G:35:ASN:HB3	2:G:50:LEU:HD23	1.88	0.55
1:C:523:CYS:O	1:C:526:LYS:NZ	2.40	0.54
3:L:76:ILE:HD13	3:L:79:LEU:HD23	1.89	0.54
1:A:136:ASP:N	1:A:136:ASP:OD1	2.41	0.54
1:A:1143:LEU:HD11	1:B:1143:LEU:HD12	1.89	0.54
1:A:332:ASN:HD21	1:A:360:VAL:HG23	1.73	0.54

Continued on next page...

For Manuscript Review

Continued from previous page...

Atom-1	Atom-2	Interatomic distance (Å)	Clash overlap (Å)
1:A:97:ASN:HB2	1:A:100:ARG:HH12	1.71	0.54
1:C:204:LYS:HE2	1:C:219:SER:HB3	1.90	0.54
1:A:333:LEU:O	1:A:359:CYS:HB2	2.08	0.54
3:L:11:LEU:HG	3:L:105:LEU:HG	1.88	0.54
3:K:3:VAL:H	3:K:26:SER:HB3	1.73	0.54
1:A:455:ARG:NH1	1:A:457:SER:O	2.41	0.53
1:B:360:VAL:HA	1:B:522:VAL:HG12	1.90	0.53
1:B:401:ARG:NH2	3:K:93:ASP:O	2.41	0.53
1:C:1082:ASP:HB2	1:C:1084:LYS:HE2	1.89	0.53
2:H:51:ILE:HD13	2:H:57:THR:HG22	1.90	0.53
1:A:336:PHE:HE1	1:A:361:ALA:HB1	1.73	0.53
1:B:394:TYR:HB2	1:B:512:SER:HB3	1.89	0.53
1:A:418:ASP:OD2	2:G:56:SER:OG	2.27	0.53
1:A:800:PHE:HB3	1:A:804:LEU:HD23	1.90	0.53
1:B:754:TYR:OH	1:B:992:ASP:OD1	2.26	0.53
1:B:1084:LYS:HE2	1:B:1120:VAL:HG11	1.90	0.53
1:C:339:VAL:HG11	1:C:354:LYS:HZ2	1.73	0.53
1:B:46:SER:HA	1:B:277:TYR:O	2.08	0.53
1:C:800:PHE:HB3	1:C:804:LEU:HD23	1.91	0.53
1:A:344:ARG:HE	1:A:507:ARG:HH22	1.56	0.53
1:A:353:ARG:HA	1:A:396:ASP:OD1	2.09	0.53
1:C:394:TYR:HB2	1:C:512:SER:HB3	1.91	0.53
1:A:351:TRP:CZ2	1:A:464:ARG:HB2	2.44	0.53
3:K:6:GLN:NE2	3:K:89:CYS:SG	2.80	0.53
1:B:127:LYS:HD3	1:B:131:PHE:HZ	1.73	0.52
1:B:104:PHE:HB2	1:B:115:LEU:HB2	1.90	0.52
2:I:68:THR:HB	2:I:81:GLN:HB3	1.91	0.52
1:A:1104:GLN:HE21	1:A:1107:PHE:HB3	1.75	0.52
1:B:328:PRO:HA	1:B:578:GLN:CD	2.30	0.52
2:I:38:ARG:NH2	2:I:89:ASP:O	2.43	0.52
1:A:895:PRO:HA	1:C:705:TYR:HE1	1.73	0.52
1:A:1079:ILE:O	1:A:1086:HIS:N	2.42	0.52
2:G:51:ILE:HG21	2:G:69:ILE:HG12	1.91	0.52
3:K:11:LEU:HG	3:K:105:LEU:HG	1.90	0.52
1:A:124:VAL:HG23	1:A:172:PRO:HA	1.92	0.52
1:A:349:TYR:HD1	1:A:451:TYR:HA	1.75	0.52
1:B:401:ARG:NH2	1:B:404:GLU:OE2	2.41	0.52
1:C:727:VAL:H	1:C:1057:GLY:HA2	1.73	0.52
1:B:127:LYS:NZ	1:B:167:GLU:OE2	2.37	0.52
2:H:47:TRP:HE1	2:H:50:LEU:HG	1.75	0.52
2:G:39:GLN:NE2	2:G:43:LYS:O	2.43	0.52

Continued on next page...

For Manuscript Review

Continued from previous page...

Atom-1	Atom-2	Interatomic distance (Å)	Clash overlap (Å)
3:J:13:LEU:HD11	3:J:105:LEU:HB3	1.92	0.52
1:A:373:PHE:N	1:A:433:ALA:O	2.42	0.52
1:B:1084:LYS:HA	1:B:1123:ASN:HA	1.91	0.52
2:H:6:GLN:HE22	2:H:95:CYS:H	1.56	0.52
2:I:4:LEU:HD12	2:I:24:ALA:HB2	1.91	0.52
1:A:396:ASP:HB2	1:A:510:VAL:HB	1.90	0.51
1:A:493:TYR:O	3:J:32:ASN:ND2	2.43	0.51
1:A:1114:THR:OG1	1:A:1116:ASP:OD1	2.28	0.51
1:C:616:THR:OG1	1:C:617:GLU:OE1	2.24	0.51
1:A:348:VAL:HB	1:A:451:TYR:HB2	1.91	0.51
1:B:325:VAL:HG22	1:B:540:ASN:HB3	1.92	0.51
1:C:137:PRO:HA	1:C:155:PHE:HA	1.93	0.51
3:J:22:SER:OG	3:J:24:ARG:NH1	2.44	0.51
1:B:135:ASN:H	1:B:157:VAL:HA	1.76	0.51
1:B:851:GLN:HB2	1:B:852:LYS:HZ2	1.76	0.51
1:B:123:ASN:HA	1:B:172:PRO:HD3	1.92	0.51
2:G:2:VAL:N	2:G:25:SER:O	2.44	0.51
2:I:53:ALA:O	2:I:71:ARG:NH2	2.44	0.51
1:C:179:GLY:HA3	1:C:184:PHE:HB3	1.92	0.51
1:C:455:ARG:NH2	1:C:465:ASP:OD2	2.43	0.51
3:K:11:LEU:HB2	3:K:105:LEU:HA	1.91	0.51
1:B:578:GLN:O	1:B:580:LEU:N	2.44	0.51
1:C:240:LEU:HD11	1:C:242:LEU:HD13	1.93	0.51
1:C:754:TYR:OH	1:C:992:ASP:OD2	2.25	0.51
2:I:45:LEU:HD11	3:K:39:GLN:HE22	1.76	0.50
1:A:334:CYS:HA	1:A:359:CYS:CB	2.39	0.50
1:C:344:ARG:NH2	1:C:440:ASP:OD2	2.44	0.50
2:H:29:VAL:HG22	2:H:76:ASN:HB2	1.92	0.50
3:K:22:SER:O	3:K:24:ARG:NH1	2.45	0.50
1:B:436:SER:OG	1:B:507:ARG:NH1	2.43	0.50
1:A:432:ILE:HG13	1:A:509:VAL:HB	1.92	0.50
1:A:465:ASP:OD1	1:A:465:ASP:N	2.44	0.50
1:B:701:ASN:ND2	1:C:785:GLN:OE1	2.45	0.50
3:J:49:VAL:HG23	3:J:55:ARG:HA	1.93	0.50
2:I:99:LEU:HG	2:I:101:GLU:H	1.76	0.50
1:A:333:LEU:C	1:A:359:CYS:HB2	2.32	0.50
1:A:351:TRP:HZ3	1:A:353:ARG:HB3	1.77	0.50
1:C:157:VAL:HG11	1:C:239:LEU:HD11	1.93	0.50
2:G:34:MET:SD	2:G:51:ILE:HG23	2.52	0.49
1:A:91:ALA:HB3	1:A:264:TYR:HB2	1.94	0.49
1:C:1114:THR:OG1	1:C:1116:ASP:OD1	2.29	0.49

Continued on next page...

For Manuscript Review

Continued from previous page...

Atom-1	Atom-2	Interatomic distance (Å)	Clash overlap (Å)
1:B:525:PRO:HB2	1:B:527:LYS:HE3	1.95	0.49
3:K:2:LEU:HD22	3:K:98:THR:HG21	1.94	0.49
1:A:452:ARG:HH22	1:A:466:ILE:HA	1.77	0.49
1:A:700:GLU:OE2	1:B:788:LYS:NZ	2.41	0.49
1:C:640:VAL:HG12	1:C:649:ILE:HG12	1.94	0.49
2:G:4:LEU:HD22	2:G:107:VAL:HG11	1.95	0.49
2:H:39:GLN:HB2	2:H:45:LEU:HD13	1.95	0.49
1:B:381:SER:HB3	1:B:384:LYS:HG2	1.95	0.49
1:B:143:TYR:HB3	1:B:146:ASN:HB3	1.94	0.49
1:B:37:TYR:OH	1:B:53:ASP:OD2	2.25	0.49
1:B:64:TRP:CD1	1:B:66:HIS:HB2	2.48	0.49
1:A:351:TRP:CZ3	1:A:353:ARG:HB3	2.47	0.48
1:A:437:ASN:HB3	1:A:504:GLN:HB3	1.94	0.48
1:B:864:THR:HG23	1:B:866:GLU:H	1.78	0.48
2:H:50:LEU:HD22	2:H:52:TYR:HE1	1.78	0.48
1:B:1095:SER:HB2	1:B:1100:TRP:CD2	2.49	0.48
1:C:981:ARG:HH12	1:C:982:LEU:HD13	1.78	0.48
1:C:499:TYR:HB3	1:C:504:GLN:HE22	1.78	0.48
1:B:288:ASP:OD1	1:B:289:CYS:N	2.47	0.48
1:A:34:ARG:HG3	1:A:214:LEU:HD11	1.95	0.48
1:C:213:ASP:OD2	1:C:264:TYR:OH	2.31	0.48
1:B:578:GLN:C	1:B:580:LEU:N	2.66	0.47
1:B:819:LEU:HD11	1:B:823:LYS:HE2	1.96	0.47
3:K:36:TRP:HB2	3:K:49:VAL:HG12	1.95	0.47
3:L:30:SER:OG	3:L:31:SER:N	2.47	0.47
1:A:287:VAL:HG21	1:A:298:LYS:HD2	1.97	0.47
1:A:327:PHE:HB3	1:A:328:PRO:HD2	1.97	0.47
1:A:329:ASN:HB2	1:A:577:PRO:O	2.14	0.47
1:A:175:MET:SD	1:A:175:MET:N	2.87	0.47
1:B:95:LYS:H	1:B:98:ILE:HD13	1.80	0.47
1:C:1084:LYS:HA	1:C:1123:ASN:HA	1.94	0.47
1:C:437:ASN:HB3	1:C:504:GLN:HB3	1.95	0.47
2:H:34:MET:HB2	2:H:51:ILE:HG23	1.96	0.47
3:L:52:ALA:HB1	3:L:66:SER:HA	1.97	0.47
1:A:348:VAL:CG2	1:A:451:TYR:HB3	2.44	0.47
1:A:904:PHE:HB3	1:A:909:VAL:HG13	1.97	0.47
1:B:455:ARG:NH1	1:B:457:SER:O	2.45	0.47
3:K:6:GLN:HG2	3:K:23:CYS:HB2	1.97	0.47
1:B:470:ILE:HD13	1:B:488:PHE:HD1	1.80	0.47
1:A:334:CYS:HB2	1:A:360:VAL:O	2.14	0.46
1:A:360:VAL:HA	1:A:522:VAL:HA	1.97	0.46

Continued on next page...

For Manuscript Review

Continued from previous page...

Atom-1	Atom-2	Interatomic distance (Å)	Clash overlap (Å)
1:B:465:ASP:OD1	1:B:465:ASP:N	2.48	0.46
2:I:4:LEU:HA	2:I:24:ALA:HA	1.97	0.46
1:A:98:ILE:HG22	1:A:240:LEU:HD11	1.98	0.46
1:A:344:ARG:HH22	1:A:439:LEU:CG	2.27	0.46
1:C:967:LYS:HE3	1:C:972:SER:HA	1.97	0.46
3:L:55:ARG:NE	3:L:63:ILE:O	2.45	0.46
2:G:3:GLN:NE2	2:G:4:LEU:O	2.48	0.46
1:A:295:SER:HA	1:A:298:LYS:HG2	1.98	0.46
1:A:349:TYR:HB3	1:A:452:ARG:H	1.80	0.46
1:A:355:ARG:HG2	1:A:393:VAL:O	2.16	0.46
1:B:603:SER:OG	1:B:604:ASN:N	2.48	0.46
1:B:1045:TYR:HB2	1:B:1065:TYR:HB3	1.97	0.46
1:C:125:VAL:HA	1:C:169:VAL:HG12	1.97	0.46
1:A:331:THR:HA	1:A:526:LYS:CE	2.45	0.46
1:A:414:GLY:H	1:A:417:ALA:HB3	1.81	0.46
1:A:768:ILE:HD11	1:A:1010:LEU:HD23	1.97	0.46
1:A:1127:VAL:HG23	1:B:915:TYR:HB3	1.97	0.46
1:C:349:TYR:HD2	1:C:452:ARG:HH21	1.64	0.46
1:C:730:THR:OG1	1:C:953:ASN:OD1	2.34	0.46
1:B:116:LEU:HD13	1:B:131:PHE:HE2	1.81	0.46
2:I:23:ALA:HB2	2:I:77:THR:HG23	1.97	0.46
1:A:101:GLY:HA3	1:A:117:ILE:O	2.16	0.46
1:B:437:ASN:ND2	1:B:504:GLN:OE1	2.43	0.46
1:C:202:TYR:HD1	1:C:223:PRO:HA	1.80	0.46
1:C:1095:SER:HB2	1:C:1100:TRP:CD2	2.51	0.46
1:B:212:ARG:NH1	1:B:214:LEU:O	2.49	0.46
1:B:969:GLY:H	1:C:753:GLN:HE22	1.62	0.46
1:C:261:ALA:N	1:C:264:TYR:HH	2.14	0.46
1:C:561:GLN:O	1:C:575:ARG:NH1	2.48	0.46
1:A:143:TYR:HB3	1:A:146:ASN:HB3	1.97	0.45
1:B:415:ASN:OD1	1:B:416:ILE:N	2.50	0.45
3:K:49:VAL:HG23	3:K:55:ARG:HA	1.98	0.45
3:L:65:GLY:HA2	3:L:74:LEU:HA	1.98	0.45
2:I:2:VAL:N	2:I:25:SER:O	2.49	0.45
1:A:362:ASP:OD1	1:A:362:ASP:N	2.44	0.45
1:B:37:TYR:OH	1:B:54:LEU:O	2.34	0.45
2:G:29:VAL:HG12	2:G:97:ARG:HD3	1.98	0.45
2:I:4:LEU:HD22	2:I:107:VAL:HG11	1.98	0.45
1:A:399:VAL:HA	1:A:507:ARG:HA	1.98	0.45
1:A:1082:ASP:O	1:A:1084:LYS:NZ	2.49	0.45
1:B:51:THR:O	1:B:272:THR:HA	2.16	0.45

Continued on next page...

For Manuscript Review

Continued from previous page...

Atom-1	Atom-2	Interatomic distance (Å)	Clash overlap (Å)
3:J:6:GLN:HG2	3:J:103:THR:HG23	1.99	0.45
1:C:119:ASN:HA	1:C:124:VAL:HG12	1.99	0.45
3:J:4:LEU:HD23	3:J:100:GLY:HA3	1.98	0.45
1:A:99:ILE:HG13	1:A:240:LEU:HD13	1.99	0.45
1:A:348:VAL:HG13	1:A:400:ILE:HD12	1.99	0.45
1:C:240:LEU:HD21	1:C:242:LEU:HD22	1.99	0.45
2:G:4:LEU:HD12	2:G:24:ALA:HB2	1.98	0.45
2:I:104:GLY:HA2	3:K:47:LEU:HG	1.97	0.45
2:H:98:ASP:HA	2:H:103:GLY:HA2	1.98	0.45
2:I:29:VAL:HG22	2:I:76:ASN:HB3	1.99	0.45
1:A:609:LEU:HD12	1:A:648:LEU:HD13	1.99	0.44
1:B:212:ARG:NH1	1:B:213:ASP:O	2.50	0.44
1:B:1070:GLU:OE1	1:B:1070:GLU:N	2.45	0.44
1:C:51:THR:O	1:C:272:THR:HA	2.18	0.44
1:C:617:GLU:HG2	1:C:618:VAL:HG23	1.99	0.44
1:C:1113:ILE:HG22	1:C:1135:VAL:HG13	1.98	0.44
3:J:26:SER:OG	3:J:27:LEU:N	2.50	0.44
1:A:54:LEU:HD12	1:A:193:LYS:HD2	1.99	0.44
1:B:350:ALA:HB1	1:B:464:ARG:HH21	1.83	0.44
1:C:409:ALA:HB3	1:C:412:GLN:HG3	2.00	0.44
2:H:64:LYS:HA	2:H:64:LYS:HD2	1.85	0.44
1:A:488:PHE:HD2	1:A:490:LEU:HD23	1.82	0.44
1:C:391:THR:HA	1:C:520:ALA:HA	2.00	0.44
1:A:360:VAL:HG22	1:A:522:VAL:HA	1.99	0.44
1:B:615:CYS:HB3	1:B:647:CYS:HB2	1.81	0.44
1:C:124:VAL:HG13	1:C:172:PRO:HA	1.99	0.44
1:C:129:CYS:SG	1:C:130:GLU:N	2.91	0.44
1:C:317:ARG:HE	1:C:317:ARG:HB3	1.53	0.44
1:C:720:VAL:HG22	1:C:1063:VAL:HG22	1.98	0.44
1:C:326:ARG:HD3	1:C:530:ASN:N	2.32	0.44
1:C:452:ARG:NH2	1:C:465:ASP:O	2.46	0.44
3:J:11:LEU:HB2	3:J:105:LEU:HA	2.00	0.44
1:A:324:ILE:HD12	1:A:531:LEU:H	1.83	0.44
1:A:345:PHE:HD2	1:A:398:PHE:C	2.21	0.44
1:C:356:ILE:HB	1:C:393:VAL:HB	1.98	0.44
2:I:38:ARG:HH21	2:I:91:ALA:HB3	1.82	0.44
1:A:1095:SER:HB3	1:A:1100:TRP:CD2	2.53	0.44
2:H:36:TRP:CH2	2:H:93:TYR:HB3	2.53	0.44
1:A:202:TYR:HB3	1:A:221:LEU:HB3	1.99	0.44
1:A:329:ASN:OD1	1:A:578:GLN:HB3	2.18	0.44
1:A:453:LEU:HB2	1:A:491:GLN:HG2	2.00	0.44

Continued on next page...

For Manuscript Review

Continued from previous page...

Atom-1	Atom-2	Interatomic distance (Å)	Clash overlap (Å)
1:A:720:VAL:HG22	1:A:1063:VAL:HG22	2.00	0.44
1:C:94:GLU:OE2	1:C:188:ARG:NH1	2.41	0.44
2:H:2:VAL:N	2:H:25:SER:O	2.51	0.44
3:J:38:GLN:HE21	3:J:85:ALA:HB3	1.83	0.44
1:A:94:GLU:OE2	1:A:188:ARG:NH1	2.43	0.43
1:A:702:SER:HB2	1:B:788:LYS:HZ3	1.83	0.43
1:C:471:TYR:HB2	1:C:489:PRO:HG3	2.00	0.43
1:A:823:LYS:HE2	1:A:823:LYS:HB3	1.86	0.43
1:C:102:TRP:HB2	1:C:117:ILE:HB	2.00	0.43
1:A:98:ILE:HG23	1:A:242:LEU:HD21	2.00	0.43
1:A:125:VAL:HA	1:A:169:VAL:HG12	2.00	0.43
1:A:349:TYR:CD1	1:A:451:TYR:HA	2.53	0.43
1:A:351:TRP:CD2	1:A:421:TYR:CD1	3.06	0.43
1:A:41:LYS:HE2	1:C:560:PHE:HD2	1.84	0.43
1:A:355:ARG:HA	1:A:393:VAL:O	2.18	0.43
1:B:326:ARG:HD2	1:B:326:ARG:HA	1.75	0.43
1:B:377:CYS:HB3	1:B:382:PRO:HG3	1.99	0.43
1:B:488:PHE:HD2	1:B:490:LEU:HD23	1.83	0.43
1:C:337:ASP:OD1	1:C:338:GLU:N	2.51	0.43
2:G:64:LYS:HD2	2:G:64:LYS:HA	1.81	0.43
1:B:401:ARG:HD2	1:B:503:HIS:CD2	2.53	0.43
1:C:983:ASP:OD1	1:C:983:ASP:N	2.51	0.43
3:J:30:SER:OG	3:J:31:SER:N	2.51	0.43
1:A:342:ALA:O	1:A:343:THR:HB	2.18	0.43
1:A:401:ARG:NH2	1:A:404:GLU:OE2	2.47	0.43
1:A:992:ASP:HA	1:A:995:ILE:HG22	2.00	0.43
1:A:1090:GLU:O	1:A:1105:ARG:NH1	2.51	0.43
1:B:63:THR:HB	1:B:65:PHE:CE2	2.54	0.43
2:G:40:ALA:HB3	2:G:43:LYS:HB3	2.01	0.43
1:B:113:GLN:HA	1:B:130:GLU:HG3	2.00	0.43
1:A:331:THR:HA	1:A:526:LYS:HE2	2.01	0.43
1:A:361:ALA:H	1:A:522:VAL:HB	1.83	0.43
1:A:1035:SER:OG	1:A:1041:CYS:SG	2.67	0.43
2:G:36:TRP:CH2	2:G:93:TYR:HB3	2.54	0.43
1:B:71:THR:O	1:B:144:HIS:NE2	2.52	0.43
1:C:30:ASN:HA	1:C:61:ASN:HA	2.00	0.43
1:A:355:ARG:HE	1:A:355:ARG:HB3	1.45	0.42
1:A:403:ASN:N	1:A:502:GLY:O	2.52	0.42
1:B:170:SER:OG	1:B:171:GLN:N	2.52	0.42
2:H:69:ILE:HD11	2:H:78:LEU:HD12	2.00	0.42
3:K:38:GLN:OE1	3:K:46:ARG:NH2	2.48	0.42

Continued on next page...

For Manuscript Review

Continued from previous page...

Atom-1	Atom-2	Interatomic distance (Å)	Clash overlap (Å)
1:B:328:PRO:HG2	1:B:330:ILE:HG12	2.01	0.42
1:B:331:THR:HG21	1:B:525:PRO:CG	2.39	0.42
1:B:564:GLY:HA2	1:C:43:PHE:HB3	2.01	0.42
1:C:110:SER:HA	1:C:130:GLU:HB3	2.00	0.42
3:J:34:LEU:HA	3:J:90:GLN:O	2.18	0.42
3:J:36:TRP:HA	3:J:88:TYR:O	2.19	0.42
1:C:554:ASN:OD1	1:C:554:ASN:N	2.52	0.42
1:B:349:TYR:HD2	1:B:452:ARG:HH21	1.66	0.42
2:H:48:VAL:HA	2:H:60:ALA:HB2	2.01	0.42
2:G:50:LEU:HD11	3:J:97:TRP:HE1	1.85	0.42
1:C:326:ARG:HD3	1:C:530:ASN:HB2	2.01	0.42
2:I:22:CYS:HB3	2:I:78:LEU:HB3	2.01	0.42
1:A:192:PHE:HD1	1:A:201:ILE:HG12	1.85	0.42
1:C:372:PHE:HB2	1:C:375:PHE:HE1	1.85	0.42
1:A:735:ASP:OD2	1:C:315:ASN:ND2	2.44	0.42
1:B:328:PRO:HB3	1:B:577:PRO:C	2.39	0.42
1:A:347:SER:HB2	1:A:350:ALA:HB3	2.00	0.42
1:B:115:LEU:HD13	1:B:128:VAL:HG22	2.02	0.42
1:C:1051:PRO:O	1:C:1052:GLN:NE2	2.48	0.42
3:L:24:ARG:NH1	3:L:71:ASP:OD1	2.53	0.42
2:G:22:CYS:HB3	2:G:78:LEU:HB2	2.01	0.42
1:A:128:VAL:HB	1:A:165:THR:HB	2.02	0.42
1:A:334:CYS:CA	1:A:359:CYS:HB2	2.49	0.42
1:A:856:LEU:HD13	1:A:957:LEU:HD22	2.01	0.42
1:A:867:MET:HB2	1:C:697:LEU:HD21	2.01	0.42
1:B:37:TYR:HB3	1:B:221:LEU:HB2	2.01	0.42
1:B:1079:ILE:O	1:B:1086:HIS:N	2.53	0.42
1:A:697:LEU:HB3	1:B:871:TYR:HE1	1.85	0.41
2:H:20:LEU:HD23	2:H:80:LEU:HD11	2.00	0.41
1:B:566:ASP:OD1	1:B:570:THR:OG1	2.35	0.41
1:B:758:CYS:HA	1:B:761:LEU:HB2	2.01	0.41
1:A:330:ILE:O	1:A:331:THR:HB	2.19	0.41
1:A:558:LEU:HB2	1:A:561:GLN:HE21	1.85	0.41
1:A:753:GLN:HE21	1:C:967:LYS:HB2	1.85	0.41
2:G:14:PRO:HA	2:G:85:LEU:HB2	2.02	0.41
1:A:334:CYS:SG	1:A:360:VAL:C	2.99	0.41
1:A:382:PRO:HA	1:A:385:LEU:HG	2.02	0.41
2:G:17:SER:HB3	2:G:81:GLN:HE21	1.86	0.41
1:A:917:ASN:O	1:A:921:ILE:HG12	2.20	0.41
1:C:189:GLU:OE2	1:C:206:THR:OG1	2.37	0.41
1:C:302:LYS:HB3	1:C:302:LYS:HE2	1.82	0.41

Continued on next page...

For Manuscript Review

Continued from previous page...

Atom-1	Atom-2	Interatomic distance (Å)	Clash overlap (Å)
1:B:334:CYS:N	1:B:360:VAL:O	2.54	0.41
1:C:275:LEU:HD23	1:C:283:ILE:HD12	2.02	0.41
1:C:330:ILE:HG22	1:C:331:THR:N	2.36	0.41
1:C:757:PHE:HD2	1:C:999:LEU:HD21	1.86	0.41
2:H:4:LEU:HD21	2:H:97:ARG:NE	2.35	0.41
3:K:65:GLY:HA2	3:K:74:LEU:HA	2.03	0.41
1:A:603:SER:OG	1:A:604:ASN:N	2.54	0.41
1:A:823:LYS:NZ	1:A:936:LEU:O	2.46	0.41
1:B:192:PHE:HD1	1:B:201:ILE:HG12	1.86	0.41
1:B:333:LEU:HA	1:B:360:VAL:O	2.20	0.41
1:B:903:ARG:NH1	1:B:1047:LEU:O	2.48	0.41
1:C:329:ASN:O	1:C:331:THR:HG23	2.20	0.41
1:C:129:CYS:HB2	1:C:164:CYS:HB3	1.81	0.41
2:G:110:GLN:NE2	2:G:111:GLY:O	2.53	0.41
1:A:1127:VAL:HG13	1:A:1130:ILE:HB	2.02	0.41
1:B:188:ARG:HB3	1:B:190:PHE:CE2	2.56	0.41
1:B:356:ILE:HD13	1:B:393:VAL:HG12	2.03	0.41
1:C:354:LYS:HE3	1:C:354:LYS:HB2	1.89	0.41
1:C:465:ASP:OD1	1:C:465:ASP:N	2.53	0.41
1:C:603:SER:OG	1:C:604:ASN:N	2.54	0.41
2:H:51:ILE:HG21	2:H:69:ILE:HG12	2.03	0.41
1:B:1137:ASP:N	1:B:1137:ASP:OD1	2.52	0.41
1:A:288:ASP:OD1	1:A:289:CYS:N	2.54	0.40
1:A:397:SER:HA	1:A:509:VAL:HA	2.02	0.40
1:C:765:LEU:HD23	1:C:765:LEU:HA	1.93	0.40
2:G:50:LEU:HB2	2:G:58:PHE:HB2	2.03	0.40
1:A:778:GLU:O	1:A:782:GLN:NE2	2.54	0.40
1:A:880:ILE:HG13	1:A:881:THR:HG23	2.03	0.40
1:C:372:PHE:HB2	1:C:375:PHE:CE1	2.57	0.40
1:A:753:GLN:NE2	1:C:967:LYS:HB2	2.35	0.40
3:K:55:ARG:HH21	3:K:64:SER:HA	1.87	0.40
1:A:553:SER:HB3	1:A:584:ASP:HB2	2.03	0.40
1:B:116:LEU:HD13	1:B:131:PHE:CE2	2.56	0.40
1:B:355:ARG:HH11	1:B:394:TYR:HE1	1.69	0.40
1:B:440:ASP:OD1	1:B:440:ASP:N	2.52	0.40
1:B:561:GLN:HA	1:C:41:LYS:HD2	2.02	0.40
1:B:817:GLU:HA	1:B:820:LEU:HD12	2.02	0.40
2:I:98:ASP:HA	2:I:103:GLY:HA2	2.04	0.40
1:A:710:ILE:O	1:A:1073:PHE:N	2.49	0.40
3:K:34:LEU:HA	3:K:90:GLN:O	2.21	0.40

There are no symmetry-related clashes.

For Manuscript Review

5.3 Torsion angles (i)

5.3.1 Protein backbone (i)

In the following table, the Percentiles column shows the percent Ramachandran outliers of the chain as a percentile score with respect to all PDB entries followed by that with respect to all EM entries.

The Analysed column shows the number of residues for which the backbone conformation was analysed, and the total number of residues.

Mol	Chain	Analysed	Favoured	Allowed	Outliers	Percentiles	
1	A	1036/1289 (80%)	956 (92%)	74 (7%)	6 (1%)	25	64
1	B	1045/1289 (81%)	978 (94%)	65 (6%)	2 (0%)	47	80
1	C	1040/1289 (81%)	963 (93%)	76 (7%)	1 (0%)	51	84
2	G	113/115 (98%)	107 (95%)	6 (5%)	0	100	100
2	H	113/115 (98%)	109 (96%)	4 (4%)	0	100	100
2	I	113/115 (98%)	107 (95%)	6 (5%)	0	100	100
3	J	105/107 (98%)	101 (96%)	4 (4%)	0	100	100
3	K	105/107 (98%)	100 (95%)	5 (5%)	0	100	100
3	L	105/107 (98%)	99 (94%)	6 (6%)	0	100	100
All	All	3775/4533 (83%)	3520 (93%)	246 (6%)	9 (0%)	50	80

All (9) Ramachandran outliers are listed below:

Mol	Chain	Res	Type
1	A	342	ALA
1	A	343	THR
1	B	579	THR
1	A	331	THR
1	C	330	ILE
1	A	359	CYS
1	B	335	PRO
1	A	328	PRO
1	A	524	GLY

5.3.2 Protein sidechains (i)

In the following table, the Percentiles column shows the percent sidechain outliers of the chain as a percentile score with respect to all PDB entries followed by that with respect to all EM entries.

For Manuscript Review

The Analysed column shows the number of residues for which the sidechain conformation was analysed, and the total number of residues.

Mol	Chain	Analysed	Rotameric	Outliers	Percentiles	
1	A	916/1115 (82%)	907 (99%)	9 (1%)	76	89
1	B	922/1115 (83%)	920 (100%)	2 (0%)	93	98
1	C	916/1115 (82%)	914 (100%)	2 (0%)	93	98
2	G	90/90 (100%)	90 (100%)	0	100	100
2	H	90/90 (100%)	90 (100%)	0	100	100
2	I	90/90 (100%)	90 (100%)	0	100	100
3	J	90/90 (100%)	88 (98%)	2 (2%)	52	78
3	K	90/90 (100%)	88 (98%)	2 (2%)	52	78
3	L	90/90 (100%)	89 (99%)	1 (1%)	73	88
All	All	3294/3885 (85%)	3276 (100%)	18 (0%)	89	95

All (18) residues with a non-rotameric sidechain are listed below:

Mol	Chain	Res	Type
1	A	212	ARG
1	A	336	PHE
1	A	337	ASP
1	A	338	GLU
1	A	340	PHE
1	A	352	ASN
1	A	353	ARG
1	A	355	ARG
1	A	360	VAL
1	B	359	CYS
1	B	578	GLN
1	C	95	LYS
1	C	212	ARG
3	L	62	ARG
3	J	24	ARG
3	J	62	ARG
3	K	24	ARG
3	K	62	ARG

Sometimes sidechains can be flipped to improve hydrogen bonding and reduce clashes. All (6) such sidechains are listed below:

For Manuscript Review

Mol	Chain	Res	Type
1	A	332	ASN
1	A	407	GLN
1	A	753	GLN
1	B	578	GLN
1	C	329	ASN
2	H	6	GLN

5.3.3 RNA (i)

There are no RNA molecules in this entry.

5.4 Non-standard residues in protein, DNA, RNA chains (i)

There are no non-standard protein/DNA/RNA residues in this entry.

5.5 Carbohydrates (i)

There are no monosaccharides in this entry.

5.6 Ligand geometry (i)

There are no ligands in this entry.

5.7 Other polymers (i)

There are no such residues in this entry.

5.8 Polymer linkage issues (i)

There are no chain breaks in this entry.

For Manuscript Review

6 Map visualisation (i)

This section contains visualisations of the EMDB entry EMD-38616. These allow visual inspection of the internal detail of the map and identification of artifacts.

Images derived from a raw map, generated by summing the deposited half-maps, are presented below the corresponding image components of the primary map to allow further visual inspection and comparison with those of the primary map.

6.1 Orthogonal projections (i)

6.1.1 Primary map

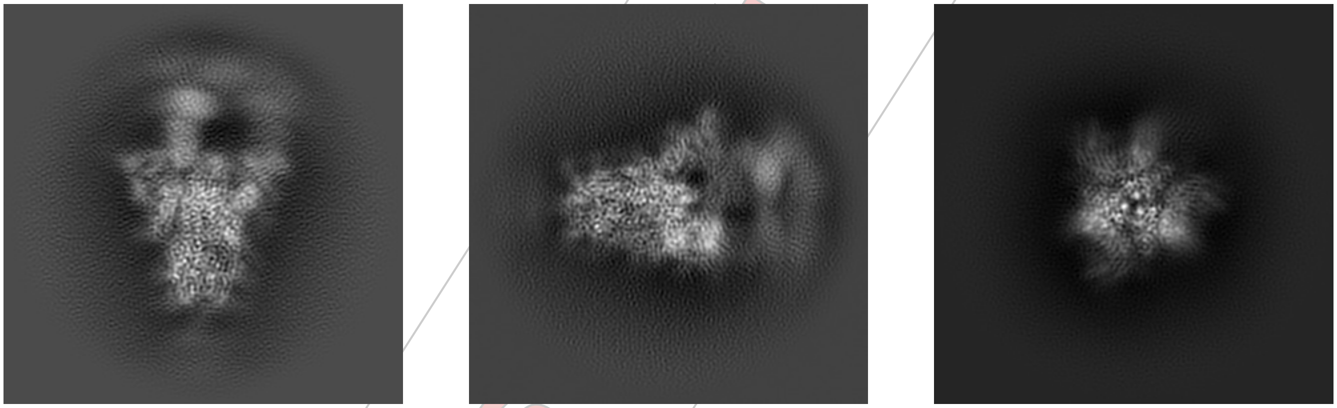

X Y Z

6.1.2 Raw map

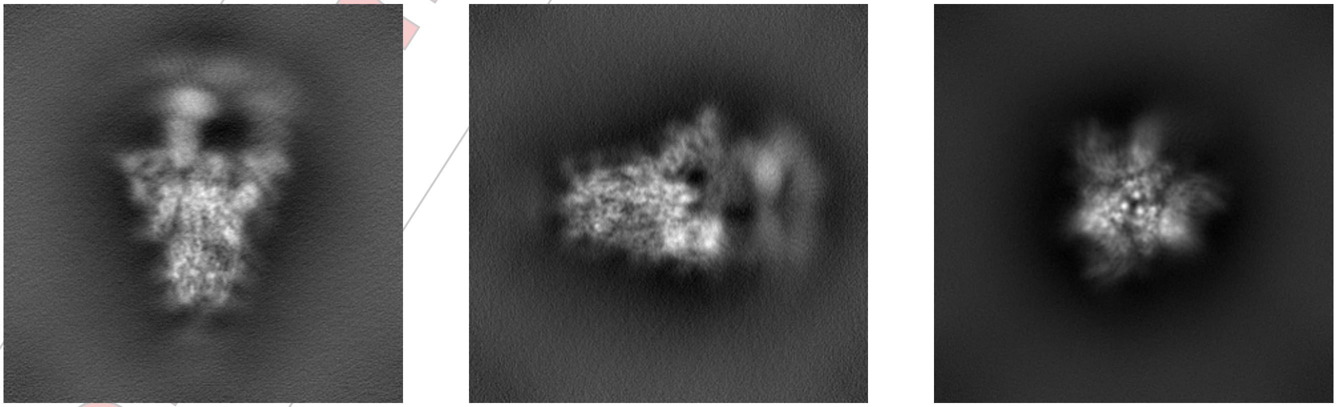

X Y Z

The images above show the map projected in three orthogonal directions.

For Manuscript Review

6.2 Central slices (i)

6.2.1 Primary map

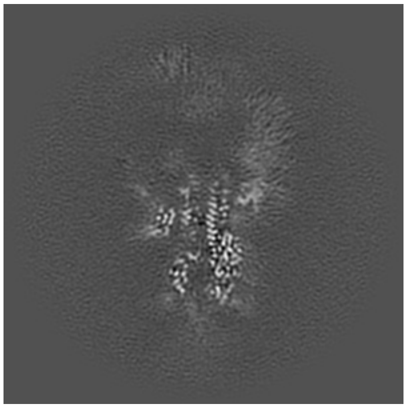
X Index: 160

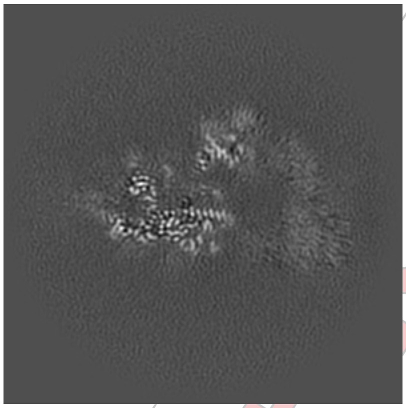
Y Index: 160

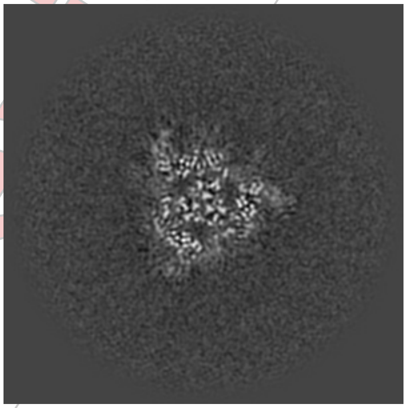
Z Index: 160

6.2.2 Raw map

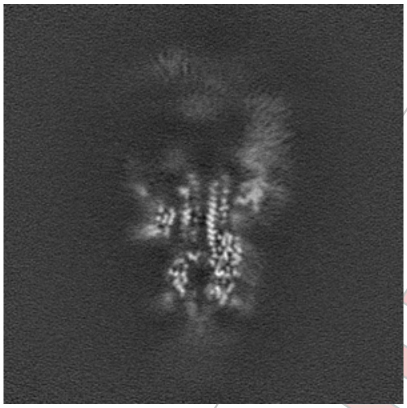
X Index: 160

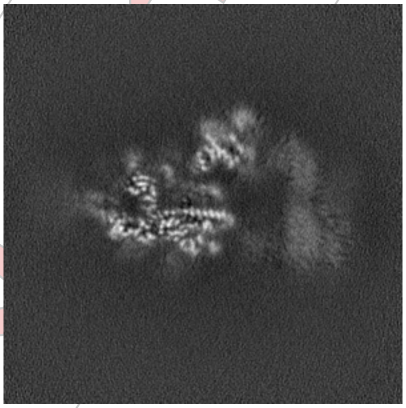
Y Index: 160

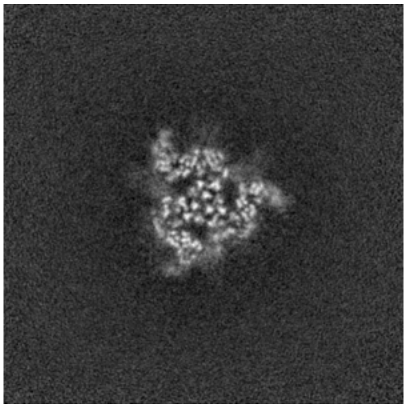
Z Index: 160

The images above show central slices of the map in three orthogonal directions.

For Manuscript Review

6.3 Largest variance slices (i)

6.3.1 Primary map

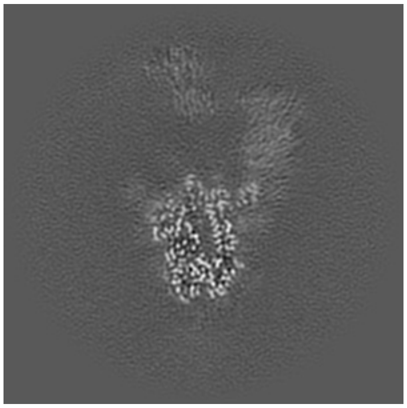
X Index: 171

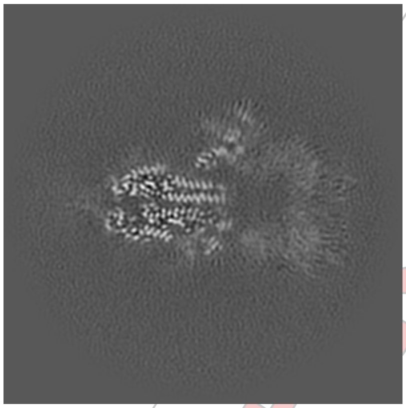
Y Index: 155

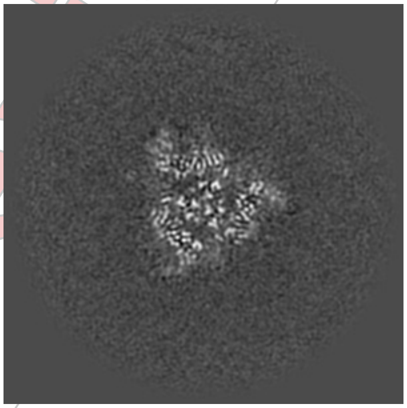
Z Index: 161

6.3.2 Raw map

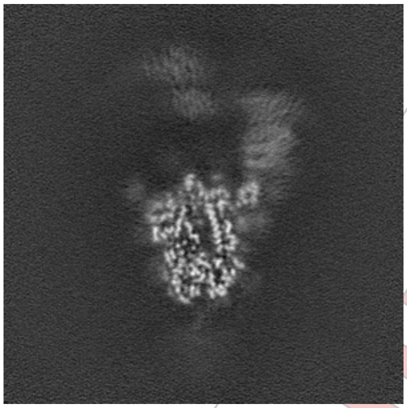
X Index: 171

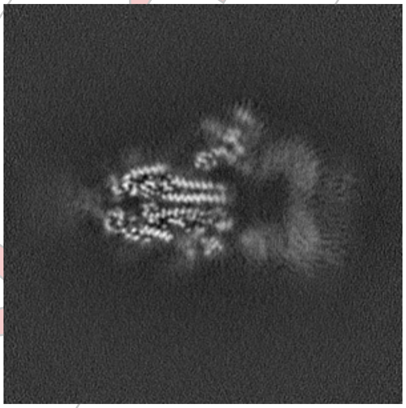
Y Index: 155

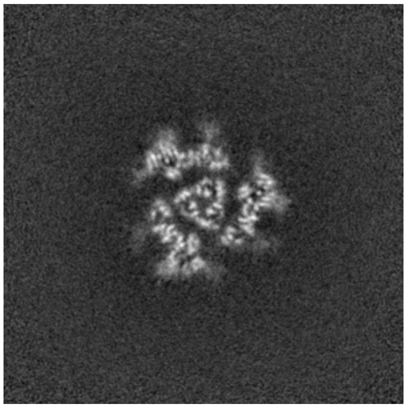
Z Index: 169

The images above show the largest variance slices of the map in three orthogonal directions.

For Manuscript Review

6.4 Orthogonal standard-deviation projections (False-color) (i)

6.4.1 Primary map

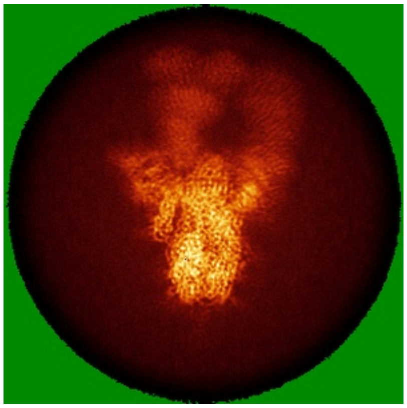
X

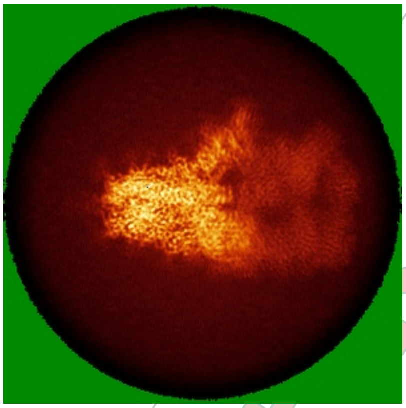
Y

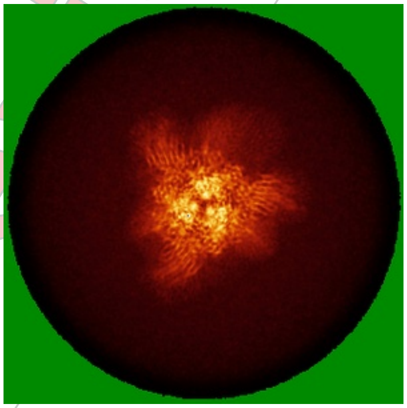
Z

6.4.2 Raw map

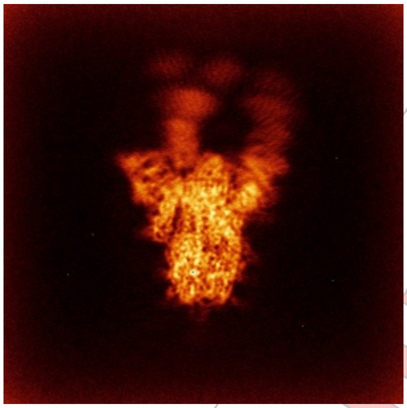
X

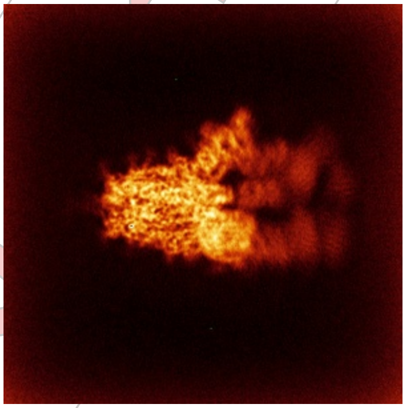
Y

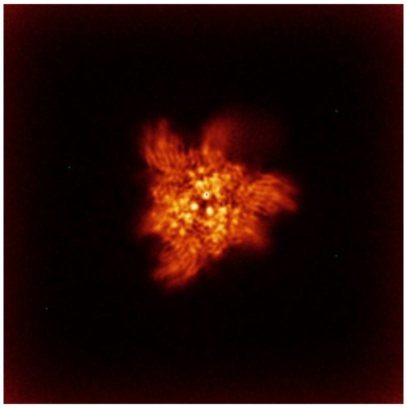
Z

The images above show the map standard deviation projections with false color in three orthogonal directions. Minimum values are shown in green, max in blue, and dark to light orange shades represent small to large values respectively.

For Manuscript Review

6.5 Orthogonal surface views (i)

6.5.1 Primary map

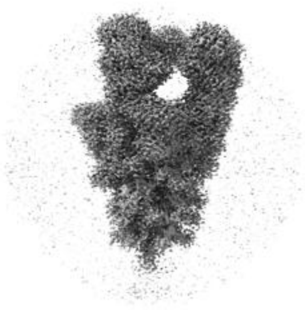

X

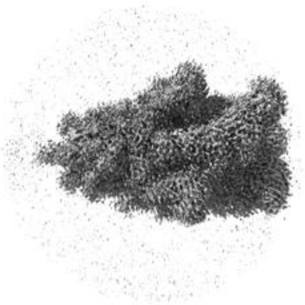

Y

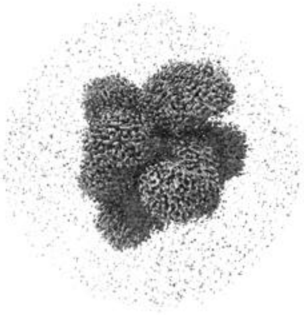

Z

The images above show the 3D surface view of the map at the recommended contour level 0.19. These images, in conjunction with the slice images, may facilitate assessment of whether an appropriate contour level has been provided.

6.5.2 Raw map

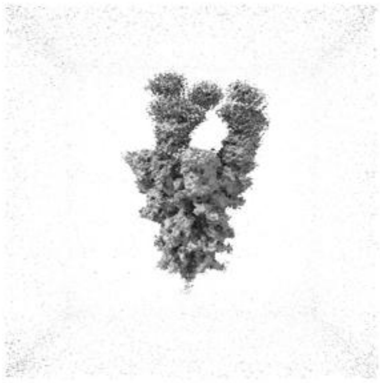

X

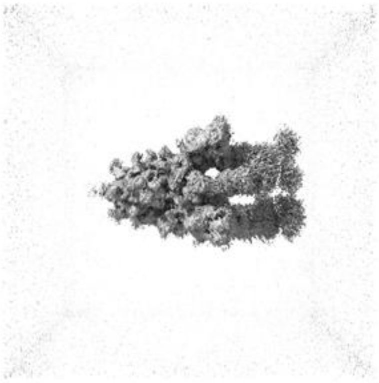

Y

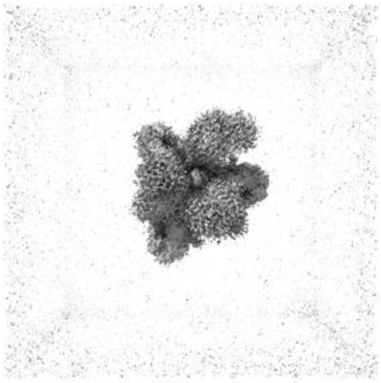

Z

These images show the 3D surface of the raw map. The raw map's contour level was selected so that its surface encloses the same volume as the primary map does at its recommended contour level.

6.6 Mask visualisation (i)

This section was not generated. No masks/segmentation were deposited.

For Manuscript Review

7 Map analysis (i)

This section contains the results of statistical analysis of the map.

7.1 Map-value distribution (i)

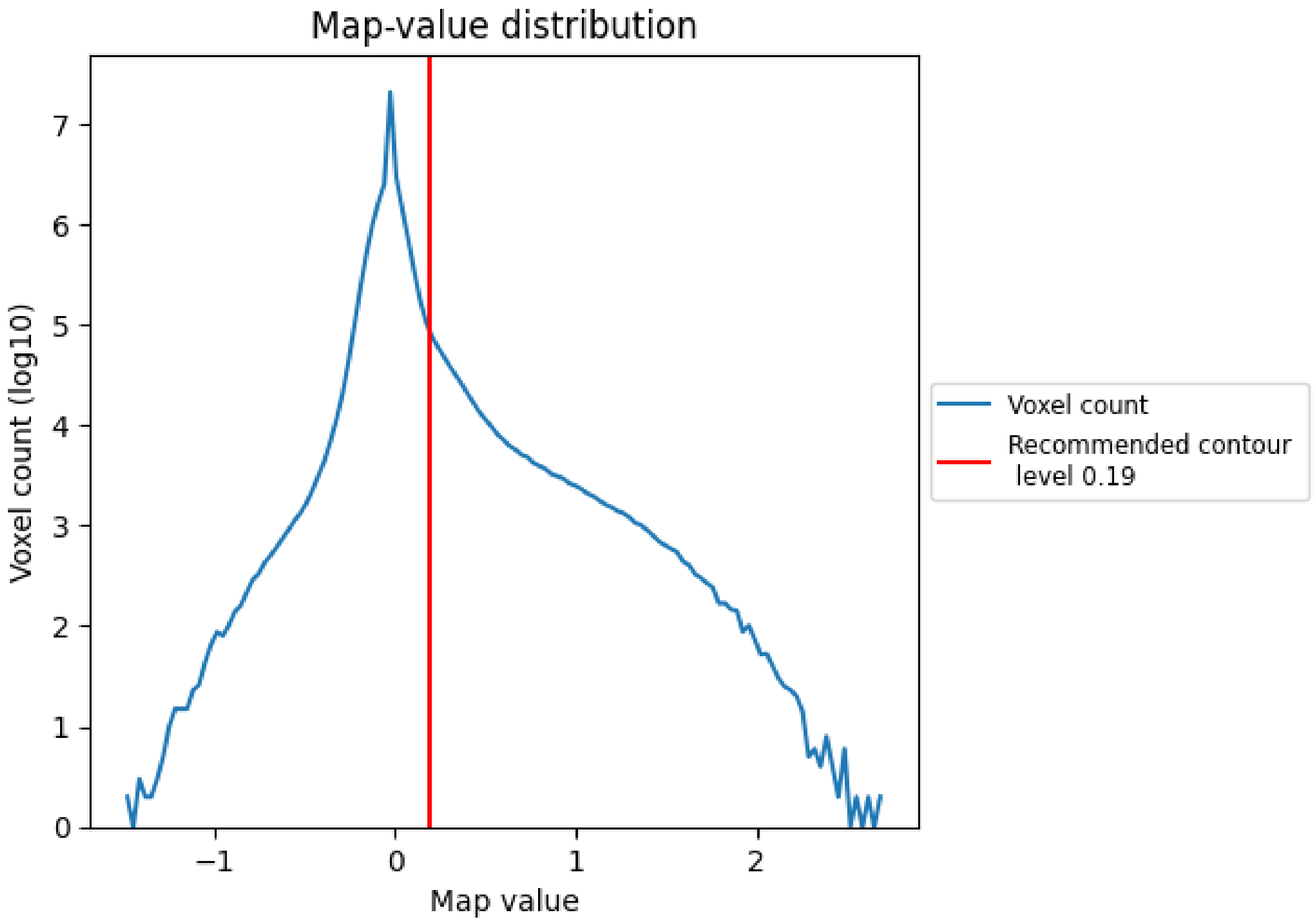

The map-value distribution is plotted in 128 intervals along the x-axis. The y-axis is logarithmic. A spike in this graph at zero usually indicates that the volume has been masked.

For Manuscript Review

7.2 Volume estimate (i)

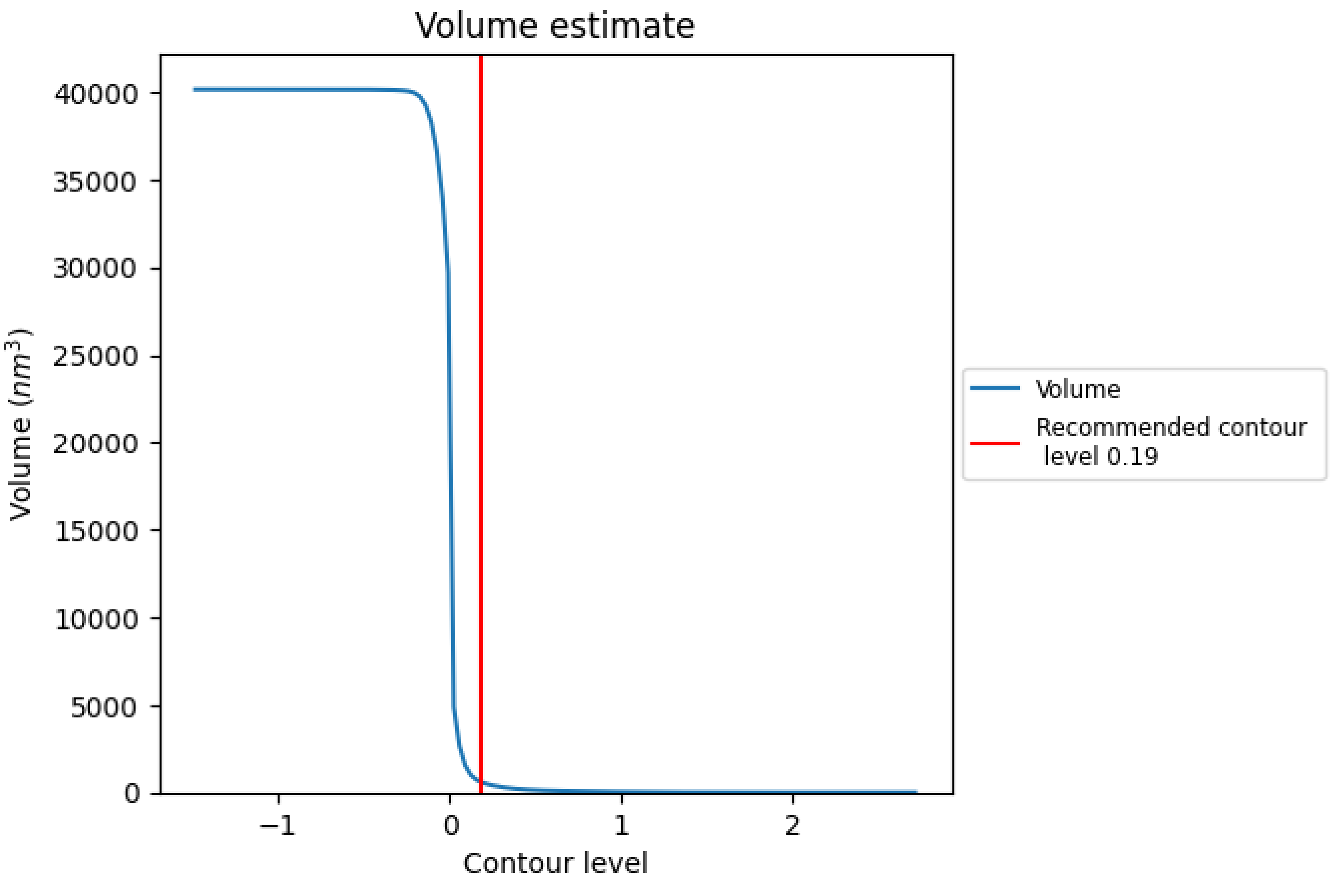

The volume at the recommended contour level is 587 nm^3; this corresponds to an approximate mass of 530 kDa.

The volume estimate graph shows how the enclosed volume varies with the contour level. The recommended contour level is shown as a vertical line and the intersection between the line and the curve gives the volume of the enclosed surface at the given level.

For Manus

7.3 Rotationally averaged power spectrum (i)

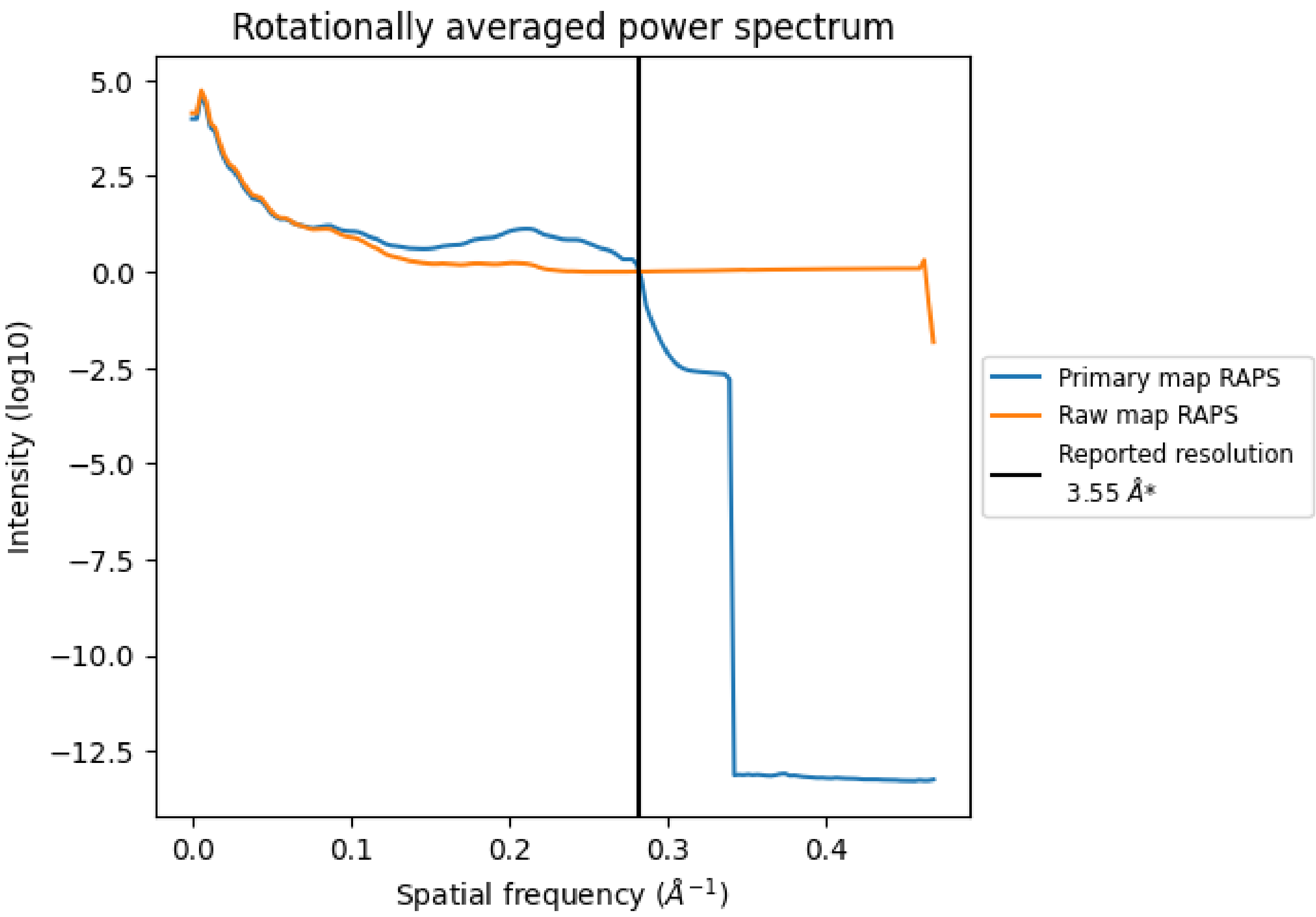

*Reported resolution corresponds to spatial frequency of 0.282 Å^{-1}

For Manuscript Review

8 Fourier-Shell correlation (i)

Fourier-Shell Correlation (FSC) is the most commonly used method to estimate the resolution of single-particle and subtomogram-averaged maps. The shape of the curve depends on the imposed symmetry, mask and whether or not the two 3D reconstructions used were processed from a common reference. The reported resolution is shown as a black line. A curve is displayed for the half-bit criterion in addition to lines showing the 0.143 gold standard cut-off and 0.5 cut-off.

8.1 FSC (i)

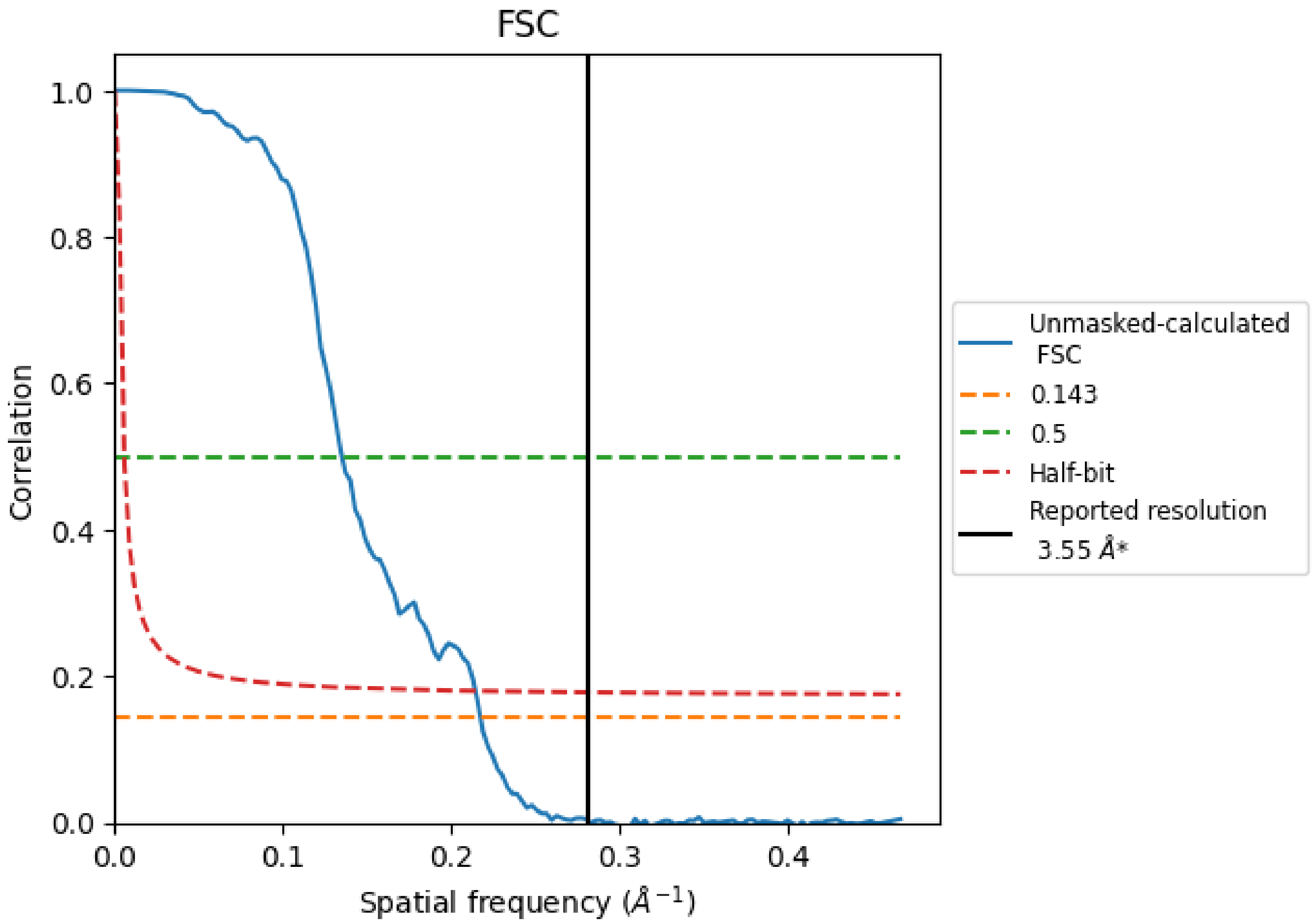

*Reported resolution corresponds to spatial frequency of 0.282 Å^{-1}

For Manuscript Review

8.2 Resolution estimates ⓘ

Resolution estimate (Å)	Estimation criterion (FSC cut-off)		
	0.143	0.5	Half-bit
Reported by author	-	-	-
Author-provided FSC curve	-	-	-
Unmasked-calculated*	4.60	7.39	4.66

*Resolution estimate based on FSC curve calculated by comparison of deposited half-maps.

For Manuscript Review

9 Map-model fit (i)

This section contains information regarding the fit between EMDB map EMD-38616 and PDB model 8XSD. Per-residue inclusion information can be found in section 3 on page 14.

9.1 Map-model overlay (i)

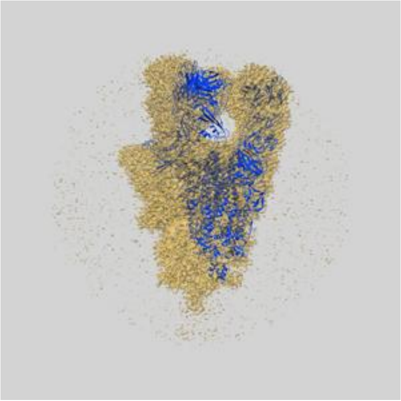

X

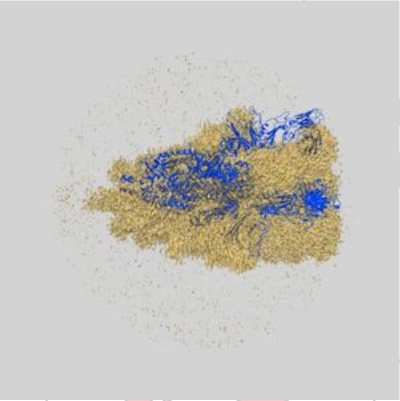

Y

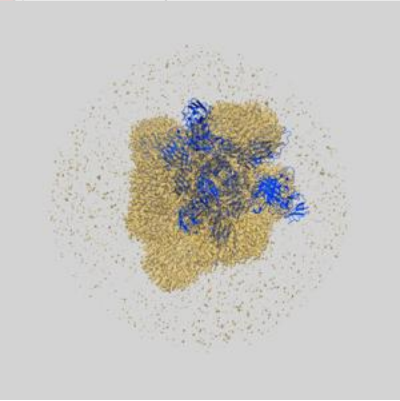

Z

The images above show the 3D surface view of the map at the recommended contour level 0.19 at 50% transparency in yellow overlaid with a ribbon representation of the model coloured in blue. These images allow for the visual assessment of the quality of fit between the atomic model and the map.

For Manuscript Review

9.2 Q-score mapped to coordinate model (i)

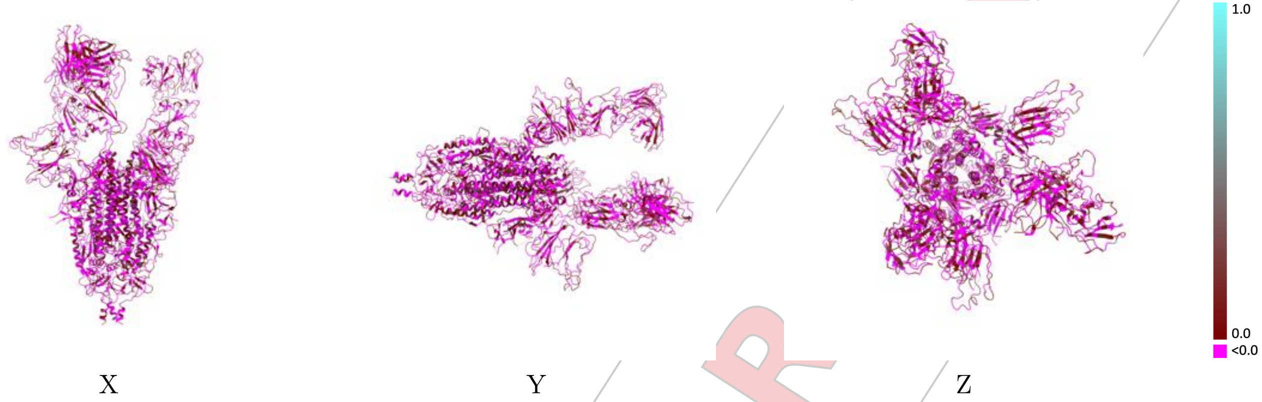

The images above show the model with each residue coloured according its Q-score. This shows their resolvability in the map with higher Q-score values reflecting better resolvability. Please note: Q-score is calculating the resolvability of atoms, and thus high values are only expected at resolutions at which atoms can be resolved. Low Q-score values may therefore be expected for many entries.

9.3 Atom inclusion mapped to coordinate model (i)

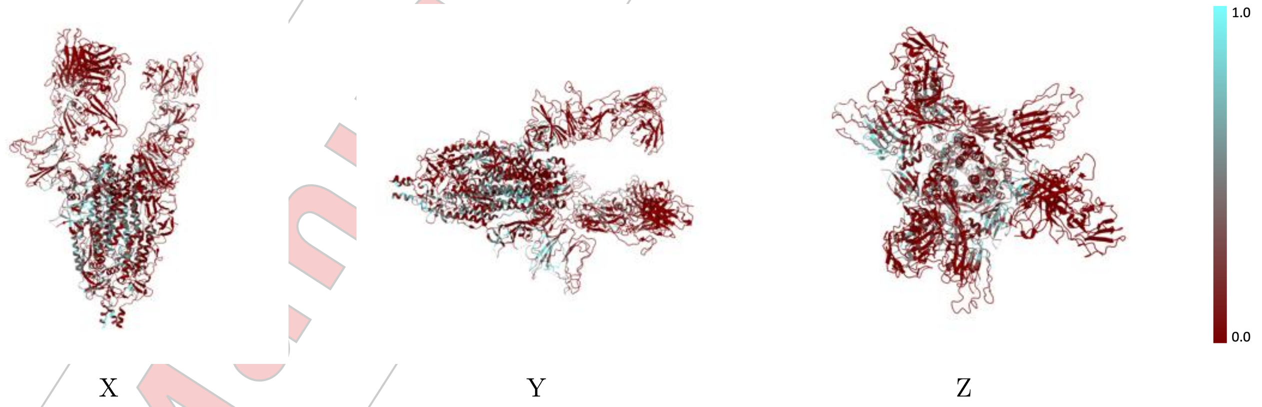

The images above show the model with each residue coloured according to its atom inclusion. This shows to what extent they are inside the map at the recommended contour level (0.19).

For Manuscript Review

9.4 Atom inclusion (i)

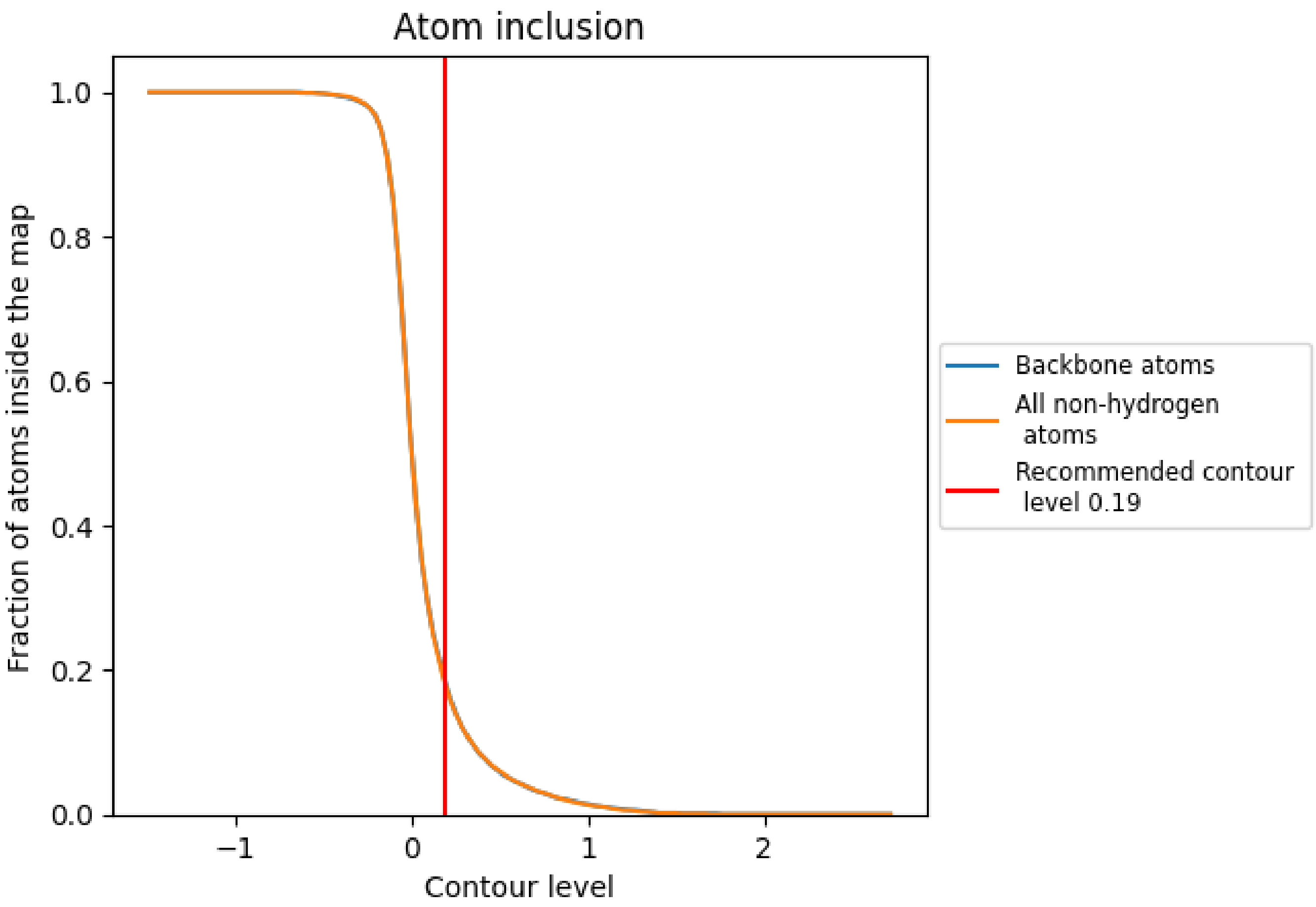

At the recommended contour level, 19% of all backbone atoms, 18% of all non-hydrogen atoms, are inside the map.

For Manuscript Review

9.5 Map-model fit summary (i)

The table lists the average atom inclusion at the recommended contour level (0.19) and Q-score for the entire model and for each chain.

Chain	Atom inclusion	Q-score
All	0.1830	-0.0080
A	0.2390	-0.0080
B	0.1410	-0.0070
C	0.2580	-0.0140
G	0.0020	0.0170
H	0.1140	0.0000
I	0.0380	-0.0250
J	0.0000	-0.0120
K	0.0060	0.0260
L	0.0620	0.0110

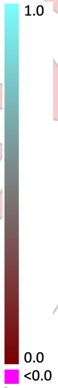

For Manuscript Review
